# Comparative transcriptomic analysis of *Porphyromonas gingivalis *biofilm and planktonic cells

**DOI:** 10.1186/1471-2180-9-18

**Published:** 2009-01-29

**Authors:** Alvin W Lo, Christine A Seers, John D Boyce, Stuart G Dashper, Nada Slakeski, J Patricia Lissel, Eric C Reynolds

**Affiliations:** 1 Cooperative Research Centre for Oral Health Science, Melbourne Dental School and the Bio21 Molecular Science and Biotechnology Institute, The University of Melbourne, 720 Swanston Street, Melbourne, Victoria, 3010, Australia; 2 Bacterial Pathogenesis Research Group, Department of Microbiology, School of Biomedical Science, Monash University, Melbourne, Victoria 3800, Australia

## Abstract

**Background:**

*Porphyromonas gingivalis *in subgingival dental plaque, as part of a mature biofilm, has been strongly implicated in the onset and progression of chronic periodontitis. In this study using DNA microarray we compared the global gene expression of a *P. gingivalis *biofilm with that of its planktonic counterpart grown in the same continuous culture.

**Results:**

Approximately 18% (377 genes, at 1.5 fold or more, *P*-value < 0.01) of the *P. gingivalis *genome was differentially expressed when the bacterium was grown as a biofilm. Genes that were down-regulated in biofilm cells, relative to planktonic cells, included those involved in cell envelope biogenesis, DNA replication, energy production and biosynthesis of cofactors, prosthetic groups and carriers. A number of genes encoding transport and binding proteins were up-regulated in *P. gingivalis *biofilm cells. Several genes predicted to encode proteins involved in signal transduction and transcriptional regulation were differentially regulated and may be important in the regulation of biofilm growth.

**Conclusion:**

This study analyzing global gene expression provides insight into the adaptive response of *P. gingivalis *to biofilm growth, in particular showing a down regulation of genes involved in growth and metabolic activity.

## Background

The gram-negative obligate anaerobe *Porphyromonas gingivalis*, in subgingival dental plaque, has been strongly implicated in the onset and progression of chronic periodontitis, a disease characterized by the destruction of the tooth supporting (periodontal) tissues [1,2]. There is increasing evidence that *P. gingivalis *is also associated with systemic diseases such as atherosclerosis [3,4] and preterm birth [4]. *P. gingivalis *is an asaccharolytic organism that relies on the catabolism of amino acids for energy production and growth [5]. An array of virulence factors has been associated with *P. gingivalis *pathogenicity, including proteases, adhesins, fimbriae and capsular polysaccharide [6,7]. The persistence of *P. gingivalis *in subgingival plaque for periods sufficiently long enough to elicit disease is inherently dependent on it surviving as part of a mature biofilm. Although mutational analyses have been employed to study genes associated with biofilm development by *P. gingivalis *[8-14], very little is known about the nature of *P. gingivalis *physiology and the crucial regulatory processes occurring in the mature *P. gingivalis *biofilm and how this relates to pathogenicity. In our laboratory we have devised a reproducible continuous culture method to grow biofilm and planktonic cells simultaneously in the same fermentor vessel. Using this approach we have compared the cell envelope proteome of *P. gingivalis *W50 biofilm and planktonic cells [15]. In this current study, we have expanded our investigation of these cells, comparing the global gene expression within *P. gingivalis *biofilm and planktonic cells using microarray analysis.

## Methods

### Continuous culture conditions and biofilm formation

The growth and physical characterization of the biofilm and planktonic cells analysed in this study have been described in Ang *et al*. [15]. The continuous culture system allows the simultaneous co-culture of planktonic cells and biofilm cells under identical growth conditions [15]. Briefly, the methods used were as follows. To produce biofilm and planktonic cells for RNA harvest *P. gingivalis *was grown in continuous culture, in duplicate, using a Bioflo 110 fermentor with a total volume of 400 mL (New Brunswick Scientific, Edison, NJ, USA) in BHI medium supplemented with 5 mg mL^-1 ^cysteine hydrochloride and 5.0 μg mL^-1 ^haemin. Growth was initiated by inoculating the fermentor vessel with a 24 hour batch culture (100 mL) of *P. gingivalis *grown in the same medium. After a 24 h incubation the media reservoir pump was turned on and the media flow adjusted to give a dilution rate of 0.1 h^-1^(mean generation time of 6.9 h). The temperature of the vessel was maintained at 37°C and the pH at 7.4 ± 0.1. The culture was continuously gassed with 5% CO_2 _in 95% N_2_. Optical density readings (OD_650 nm_) and purity of the culture were analyzed daily. Biofilm could be seen to be forming on the fermentor vessel walls and on glass microscope slides that were fixed to the vessel walls. Each *P. gingivalis *W50 culture was maintained for 40 days until harvesting. Planktonic cells were harvested by rapidly pumping them out of the fermentor vessel. The microscope slides were then removed from the fermentor vessel for examination of biofilm thickness and cell viability. The biofilm was rinsed twice with cold PGA buffer [16] to remove contaminating planktonic cells and then removed by scraping with a spatula and suspended in cold PGA buffer in a 50 mL centrifuge tube. PGA buffer contained 10.0 mM NaH_2_PO_4_, 10.0 mM KCl, 2.0 mM citric acid, 1.25 mM MgCl_2_, 20.0 μM CaCl_2_, 25.0 μM ZnCl_2_, 50.0 μM MnCl_2_, 5.0 μM CuCl_2_, 10.0 μM CoCl_2_, 5.0 μM H_3_BO_3_, 0.1 μM Na_2_MoO_4_, 10 mM cysteine-HCl with the pH adjusted to 7.5 with 5 M NaOH.

### Biofilm characterization

The viability of cells comprising the biofilms that were on the glass microscope slides were determined using LIVE/DEAD^® ^BacLight™ stain as per manufacturer instructions (Invitrogen) with visualized using confocal laser scanning microscopy (CLSM) essentially as described by Loughlin *et al*. [17]. CLSM was done using an Axiovert 200 M inverted microscope (Carl Zeiss Pty Ltd. Germany) fitted with a Zeiss LSM 510 META Confocal scan head. Imaging was carried out using the 458/477/488 nm Argon and 543 nm HeNe laser lines and a 63× C-Apochromat^® ^water immersion lens. Live and dead cells in the stained biofilms were quantified using COMSTAT software [18] with the viability of the biofilm obtained by averaging the number of live cells over the entire z-stack [15]. Biofilm thickness was also measured using light microscopy [15].

### Total RNA extraction

*P. gingivalis *W50 biofilm and planktonic samples (40 mL) were immediately added to 0.125 volume of ice-cold Phenol solution (phenol saturated with 0.1 M citrate buffer, pH 4.3, Sigma-Aldrich, Inc. Saint Louis, MO). The mixture was centrifuged and the pellet suspended in 800 μL of ASE lysis buffer (20 mM Na acetate, 0.5% SDS, 1 mM EDTA pH 4.2) and transferred into a 2 mL microcentrifuge tube. An equal volume of ice cold Phenol solution was added and the mixture was vortexed for 30 s before incubation at 65°C for 5 min. The mixture was then chilled on ice for 3 min after which of 200 μL of chloroform was added and mixed by brief vortexing. The mixture was centrifuged at 16,100 × g and the aqueous phase collected and extracted using a Phenol solution/chloroform (1:1 vol:vol) mix. The RNA in the aqueous phase was precipitated by addition of 700 μL of 4 M LiCl and incubated overnight at -20°C. Samples were then thawed and the total RNAs were pelleted by centrifugation. The pellet was washed with cold 70% ethanol, air dried and suspended in 50 μL of 0.1% diethylpyrocarbonate treated water. The samples were then treated with DNase I (Promega, Madison, WI) and purified using RNeasy Mini columns (Qiagen, Valencia, CA) according to protocols supplied by the manufacturer. The quality of the total RNA was verified by analytical agarose gel electrophoresis and the concentration was determined spectrophotometrically.

### Microarray analyses

Reverse transcription reactions contained 10 μg of total RNA, 5 μg of random hexamers, the first strand buffer [75 mM KCl, 50 mM Tris-HCl (pH 8.3), 3 mM MgCl_2_], 0.63 mM each of dATP, dCTP, and dGTP, 0.31 mM dTTP (Invitrogen Life Technologies, Carlsbad, CA) and 0.31 mM aminoallyl dUTP (Ambion, Austin TX), 5 mM DTT, and 800 u of SuperScript III reverse transcriptase (Invitrogen). The reaction mixture was incubated at 42°C for 2 h. The RNA was hydrolysed by incubation with 0.5 M EDTA and 1 M NaOH at 65°C for 15 min and the sample neutralized with 1 M HCl before purification of the cDNA with QIAquick columns (Qiagen). The cDNAs were coupled with monoreactive Cy3 or Cy5 (40 nmol) (Amersham Biosciences, Piscataway, NJ) in the presence of 0.1 M NaHCO_3 _for 60 min at room temperature. The labeled cDNAs were purified using QIAquick columns (Qiagen), combined and vacuum dried. Samples were then suspended in hybridization buffer containing 50% formamide, 10× SSC (150 mM sodium citrate, pH 7.0 and 1.5 M NaCl), 0.2% SDS and 1 μg μL^-1 ^salmon sperm DNA.

*P. gingivalis *microarrays were kindly provided by The Institute for Genomic Research (TIGR) (now The J. Craig Venter Institute). Each microarray consisted of 1907 70-mer oligonucleotides spotted in quadruplicate on a glass slide (CMT-GAPS; Corning, Corning, N.Y.). Detailed array information can be viewed at http://www.tigr.org and http://www.brop.org. A total of four slides were used for each planktonic-biofilm pair, where the cDNAs were labeled with the alternative dye and hybridized to the microarray slides using a dye-swapping design.

Slides were prehybridized at 42°C in 5× SSC, 0.1% SDS and 2% bovine serum albumin for 2 h and then briefly rinsed with distilled water and isopropanol. Slides were dried by centrifugation for 3 min at 1,500 × g. The labeled cDNAs hybridization mix was heated to 100°C for 2 min before adding to the DNA microarray. Each array was covered with a coverslip and placed inside a hybridization chamber (Corning Incorporated Life Sciences, Acton, MA). Hybridization was carried out in a 42°C water bath for approximately 16 h after which the coverslips were removed and the slides washed in 2× SSC, 0.1% SDS at 42°C. The arrays were washed at room temperature once with 0.1× SSC, 0.1% SDS for 10 min, four times for 1 min in 0.1× SSC, and then rinsed with distilled water followed by 100% ethanol. The arrays were dried immediately by centrifugation (3 min, 1,000 × g).

### Image and data analysis

The hybridized arrays were scanned using an Agilent G2565AA microarray scanner system (Agilent Technologies, Santa Clara, CA). Imagene 6.0 software (Biodiscovery, Los Angeles, CA) was used for spot finding, signal-background segmentation, and intensity quantification. The intensity of each spot was local background corrected using GeneSight 4.1 (Biodiscovery) and the resultant data were log transformed such that the mean value for each channel (Cy3 and Cy5) had a log ratio of zero. The signal intensities for each dye swap hybridization were combined and the average log ratios were used for all further analysis. The data were normalized using intensity dependent Lowess normalization [19] per spot and per slide to remove the intensity-dependent deviation in the log_2 _(ratio) values. Identification of differentially regulated genes was performed using the GeneSight 4.1 confidence analyzer [based on an ANOVA approach of Kerr et al [20]]. This statistical analysis uses replicate spots to estimate an empirical distribution of noise. The constructed noise model is then used to determine the statistical measures for the likelihood of false positives above or below a certain expression ratio. The differentially regulated genes were identified at 99% confidence intervals with a cut-off value of log_2 _> 0.6 or log_2 _< -0.6. These values correspond to approximately 1.5 fold up- and down-regulated genes, respectively, a ratio considered biologically relevant [21,22]. The stringent 99% confidence interval was selected to reduce the chance of false positive genes to 1% (*P*-value < 0.01). All DNA microarray work in this study was in compliance with MIAME guidelines and all data have been deposited under accession number E-TABM-467, in the ArrayExpress databases http://www.ebi.ac.uk/arrayexpress.

### Validation of microarray data by real time, reverse transcription-PCR

Total RNA (1 μg) was reverse transcribed to cDNA using SuperScript III First Strand Synthesis Supermix (Invitrogen) in the presence of random primers (50 ng) according to the manufacturer's recommendations. Real time-PCR was carried out using a Rotor-Gene 3000 (Corbett Research, Sydney, Australia). The primers for the real-time analysis (Table 1) were designed using Primer3 software http://primer3.sourceforge.net/. The lengths of the primers were 18 to 20 nucleotides and the amplified products between 109 and 130-bp. The amplification efficiency of each primer set was determined empirically by using cDNA template dilutions over four orders of magnitude. The amplification efficiency for each primer set varied between 95.4% and 106.6%, showing that the amplicons were generated with comparable efficiency.

**Table 1:** Primers used for real-time reverse transcription PCR

Gene ID	Forward primer 5'-3'	Reverse primer 5'-3'
PG0158	TTCTTTTGGTGGACGATGTG	GAGGGACGCTTGGTAACG
PG0270	TCGCAAGCCAAGCAAATAC	GAGATAGGGTGCGATGGTTG
PG0347	TCGGCGATGACTACGACA	CGCTCGCTTTCTCTTCATTC
PG0553	CCGATGGCAATACGAGCCGC	ATAGCCGGGGCACAGAGGGC
PG0593	CAAAAGGTCGCTCCACTCA	GTTCGCCACGATCATTCAC
PG0914	TCATCGCTCGCAGTAAGAAC	CTGAATACCGAATCCCCATC
PG1055	AGCCAACAGGAGATGGAGTG	TCAAGTCGGAGTGCGAAAA
PG1431	CGCAGACCAATCGCATAAG	CAGAATAGCCATCGCACAGA
PG1432	CCATGCAGCAAGGAGATACA	TAGTGTCGAGGGCCATTTTC

The real time-PCR reaction contained 12.5 μL of Platinum SYBR Green qPCR SuperMix-UDG (Invitrogen), 0.2 μM of each gene-specific primer and 5 μL of cDNA template. The cycling conditions were 50°C for 2 min, 95°C for 2 min, then 40 cycles of 95°C for 15 s, 58°C for 30 s, and 72°C for 30 s. Negative controls of distilled water and total RNA samples were included in each run. All reactions were carried out in triplicate and melting curve analysis indicated that in each reaction a single product was amplified.

PG0347 encoding a putative UDP-glucose 4-epimerase, *galE*, was selected as normalizer for all reactions. The critical threshold cycle, C_T _for each gene was generated by the Rotor-Gene 6 software (Corbett Research) and the relative expression ratio of the selected genes calculated and analyzed using the relative expression software tool (REST) http://www.gene-quantification.info[23]. Each real time-PCR reaction was performed using the biological replicate total RNA samples that were used for microarray analysis.

## Results and Discussion

### *P. gingivalis *W50 growth in continuous culture and biofilm formation

*P. gingivalis *is a slow growing anaerobe that even in rich media has a generation time of 4.65 h [24]. In the continuous culture system we employed here *P. gingivalis *W50 replicated with a mean generation time of 6.9 h and reached steady state approximately 10 days after inoculation. The cell density of the culture remained constant, after it had reached steady state, at an OD_650 nm _of 2.69 ± 0.21 and 2.80 ± 0.52 for the first and second biological replicates respectively. Robust biofilm was obtained on the vertical surfaces of the fermentor vessel walls and at 40 days of culture the planktonic and biofilm cells from the fermentor vessel were harvested for analysis. The glass microscope slides that were fixed to the fermentor vessel walls were used for physical characterization of the biofilm. CLSM revealed that the surface of the biofilm featured variable structures and the average percentage of viable cells within the biofilm was 91.2 ± 7.3% [15]. The biofilms were on average 240 ± 88 μm thick. Our continuous culture system allowed us to obtain a direct paired comparison of transcriptomic profiles of both the planktonic and biofilm grown cells that were cultivated in the same fermentor vessel and therefore were subjected to identical gross environmental influences (such as media composition and temperature).

### Identification of genes differentially regulated during biofilm growth

Microarray hybridizations were conducted using the paired planktonic cell and biofilm total RNA samples obtained from the two independent continuous cultures. For each culture planktonic cell and biofilm pair, four technical replicates of array hybridizations were performed (2 array slides for each dye swap) yielding 16 measurements per gene as each gene was represented in quadruplicate on each slide. We designated all genes with an average expression ratio of 1.5-fold (up or down) differentially regulated, a threshold reported to be biologically significant [21,22]. Moreover, we used the GeneSight 4.1 (Biodiscovery) confidence analyzer to discriminate genes that had a 99% likelihood of being differentially regulated at above or below the 1.5 threshold.

A total of 561 and 568 genes were identified to be differentially regulated (1.5 fold or more, *P*-value < 0.01) between the biofilm and planktonic cells of the first and second replicates respectively (data not shown). Of the identified genes, 377 belonged to a common data set (67% and 66% of the total genes identified for the first and second replicates respectively). Of the 377 genes in the common dataset 191 were up-regulated and 186 were down-regulated (see Additional files 1 and 2). This represents approximately 18% of the *P. gingivalis *genome.

**Figure 1:**
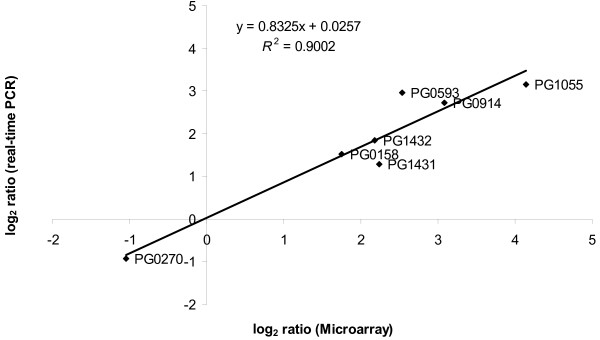
**Correlation between microarray and real-time PCR**. Correlation between microarray and real-time-PCR gene expression ratios determined for biofilm versus planktonic cells. The log_2_-transformed microarray and real-time-PCR ratios were used to determine the Spearman Rank correlation coefficient (r = 0.86, p < 0.01).

To validate the microarray data real time-PCR of selected genes PG0158, PG0270, PG0593, PG0914, PG1055, PG1431 and PG1432 was performed. Six of the genes were selected from the up-regulated group and one from the down-regulated group in biofilm cells. The expression of *galE *was detected to remain unchanged during biofilm and planktonic growth (data not shown) and was used for normalization. There was a high correlation between the expression ratios determined by both methods (R^2 ^= 0.9002) (Fig. 1).

Although in some studies the differential expression of only a small percentage of the genome has been suggested following comparison of gene expression in biofilm and planktonic cells [25-28] differential expression of a large number of genes has been demonstrated in other studies. For example, in *Escherichia coli*, using gene-fusion studies, 38% (out of 446 clones) underwent altered expression during biofilm development [29]. Sauer and co-workers demonstrated that more than 50% (over 800 proteins) of the *Pseudomonas aeruginosa *proteome was differentially regulated between planktonic growth and the fully mature biofilm [30]. Moreover, DNA microarray analysis indicated that up to 22% (a total of 580 genes) of the *Staphylococcus aureus *genome underwent expression changes during mature biofilm growth [31].

Factors shown to be relevant to *P. gingivalis *homotypic biofilm formation and heterotypic biofilm formation with *Streptococccus gordonii *include InlJ, an internalin family-related protein [13], the minor fimbrial protein MfaI [32], ClpXP [33] and the low molecular weight tyrosine phosphatase Ltp1 [34]. In the sequenced *P. gingivalis *strain W83 [35] and in our laboratory strain W50 (data not shown) the *mfa1 *gene encoding Mfa1 has been interrupted by an insertion of the mobile element IS*Pg*4. The microarray data indicated that in strain W50 biofilm cells there was increased expression of PG0176 which is the 5-prime region of *mfa1*. Thus there is an indication that in *P. gingivalis *strains where *mfa1 *is intact and Mfa1 produced that the minor fimbrillin may be upregulated in a biofilm. *P. gingivalis *coaggregation with *S. gordonii *mediated by MfaI is suggested to be relevant to *P. gingivalis *host colonizaton [36]. Increased Mfa1 production may in some strains improve host colonization, but for strains such as W50 it would not play a role in their pathogenesis. Differential expression of the genes encoding InlJ (PG0320) and ClpXP (PG0417, PG0418) was not observed in the current study.

**Figure 2:**
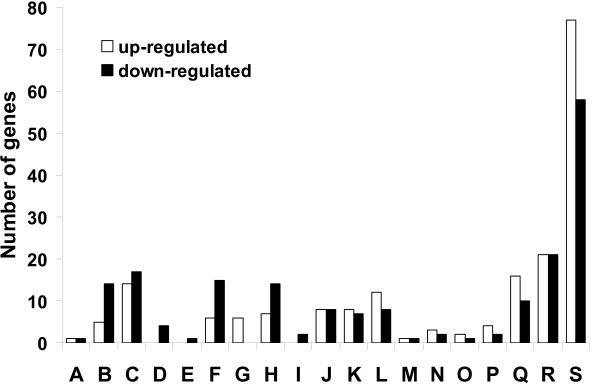
**Genes differently expressed in *P. gingivalis *W50 biofilm**. Genes differentially expressed in *P. gingivalis *W50 biofilm cells relative to planktonic cells (1.5 fold or more, *P*-value < 0.01) grouped by TIGR functional role categories. A, amino acid biosynthesis; B, biosynthesis of cofactors, prosthetic groups, and carriers; C, cell envelope; D, cellular processes; E, central intermediary metabolism; F, DNA metabolism; G, disrupted reading frame; H, energy metabolism; I, fatty acid and phospholipid metabolism; J, mobile and extrachromosomal element functions; K, protein fate; L, protein synthesis; M, purines, pyrimidines, nucleosides and nucleotides; N, regulatory functions; O, signal transduction; P, transcription; Q, transport and binding proteins; R, unknown function; and S, hypothetical or conserved hypothetical proteins.

The predicted cellular roles of the differentially regulated *P. gingivalis *gene products in this study encompass widespread functional categories (Fig. 2). However, 40% (77) of the up-regulated genes and 31% (58) of the down-regulated genes were annotated as encoding hypothetical or conserved hypothetical proteins. Genes encoding proteins with similarity to experimentally identified proteins with unknown functions accounted for about 10% of the differentially expressed genes.

### The physiology of the biofilm

The down-regulation of many genes involved in cell envelope biogenesis, biosynthesis of cofactors, prosthetic groups and carriers and other cellular processes was observed in this study (Fig. 2). Similarly, many genes involved in energy production, DNA replication, fatty acid and phospholipid metabolism and central intermediary metabolism were also down-regulated. Taken together, these observations suggest a down-turn in cell replication and a slowed growth rate in biofilm cells.

The primary indication of the slowing of cell replication in the biofilm was the down-regulation of genes encoding proteins involved in chromosome replication such as DnaA (PG0001), the primosomal protein n' PriA (PG2032), single-stranded binding protein Ssb (PG0271), the DNA polymerase III alpha subunit DnaE (PG0035) and the DNA polymerase III beta subunit DnaN(PG1853). Also down-regulated in biofilm cells were genes encoding homologues of proteins involved in DNA repair and recombination, MutS [37] (PG0412), *radA *[38] (PG0227) and *recN *[39,40] (PG1849). The biofilm cells also displayed up-regulation of a putative translational regulator, RecX (PG0157) that in *E. coli *has been shown to inhibit RecA activity which is important in homologous recombination and in the SOS pathway of DNA repair and mutagenesis [41].

The down-regulation of a significant number of genes associated with cell envelope biogenesis (see Additional files 1 and 2) also suggests that the growth rate was reduced in biofilm cells. The slower growth rate of cells in a biofilm has been previously attributed to restricted penetration of nutrients and helps explain the relative resistance of biofilms to antibiotics targeting growth [42,43]. As biofilm cells exhibit a slower growth rate then the need for energy would decrease correspondingly. Indeed, the transcriptomic data showed that expression of seven genes involved in the glutamate catabolism pathway, one of the key sources of energy for *P. gingivalis *[44], were simultaneously down-regulated in biofilm cells. One down-regulated gene in this pathway was PG1812 which is predicted to encode the alpha subunit of 2-oxoglutarate oxidoreductase, an enzyme located at the branching point in this pathway between butyrate and propionate end-products. Three genes PG0690, PG1075 and PG1076 encoding 4-hydroxybutyrate CoA-transferase, the coenzyme A transferase beta subunit and acyl-CoA dehydrogenase (short-chain specific) respectively, that are in the pathway branch that produces butyrate, were down-regulated, as were a cluster of genes encoding a methylmalonyl-CoA decarboxylase (PG1608-1612) that is part of the pathway branch that produces propionate.

### Signal transduction, regulatory and transcription genes

It has been well established that two-component signal transduction systems (TCSTSs) play an important role in biofilm formation in many bacteria, including *E. coli *[45], *Enterococcus faecalis *[46] and *Streptococcus mutans *[47]. Interrogation of the *P. gingivalis *W83 ORFs revealed only 6 putative TCSTSs. The transcriptomic analysis indicated that one of these TCSTSs, comprising PG1431 and PG1432, that encode a DNA-binding response regulator of the LuxR family and a putative sensor histidine kinase respectively, was up-regulated in biofilm cells. To date, the involvement of signal transduction, transcriptional regulators and other transcription factors in *P. gingivalis *biofilm development has yet to be established.

Homologues of the TCSTSs PG1431 and PG1432 have been found in *P. gingivalis *strain ATCC 33277 and were designated *fimR *and *fimS*, respectively [48]. FimR and FimS are known to regulate FimA-associated fimbriation [48]. Comparative transcriptomic profiling of *P. gingivalis *ATCC 33277 and its *fimR *deficient mutant indicated only a limited number of genes were part of the *fimR *regulon including PG1974, PG0644 (*tlr*) and the first gene of the *fim *locus, PG2130 [49]. Binding of FimR upstream of PG2130 initiates an expression cascade involving PG2131-34. The transcriptomic data presented here do concur with the possible positive regulation of PG1974 by PG1431, however, they are in conflict with the role of PG1431 in the positive regulation of the *fim *locus because in strain W50 biofilms we observed decreased expression of PG2133 and PG2134 with no differential expression of *fimA*. Thus, the role of PG1431 and PG1432 in *P. gingivalis *W50 biofilm growth may not be reflected in the earlier study of *P. gingivalis *ATCC 33277 FimS and FimR mutants.

It is predicted that there are 29 orphan transcriptional regulatory proteins in *P. gingivalis *but only 4 of these were differentially regulated in biofilm cells, one of which was the down-regulated PG0270, *oxyR*. The remaining three possible transcriptional regulators PG0173, PG0826 (of the AraC family of transcriptional regulators) and PG2186 were found to be up-regulated. Members of the AraC family of transcriptional regulators have been shown to be important in carbon metabolism, stress response and virulence in other species (for review see Gallegos), [50] and in the regulation of quorum sensing signaling in *P. aeruginosa *[51].

Sigma factors are the subunit of RNA polymerase responsible for the recognition of the specific sequence of the target gene promoter [52] and are involved in the regulation of diverse physiological processes, particularly virulence [53,54] and biofilm formation [55,56]. The array data indicated that three putative sigma factors of the σ^70 ^family PG0594 (*rpoD*), PG1660 and PG1827 were differentially regulated in biofilm cells. Both PG0594 and PG1660 were up-regulated whilst PG1827 was down-regulated in biofilm cells. The observed differential expression of these sigma factors in biofilm cells may indicate that these proteins are important regulators of *P. gingivalis *during biofilm growth.

### Genes encoding transport and binding proteins

Many genes predicted to encode transport and binding proteins were up-regulated in biofilm cells (Fig. 2). Six of these genes encode components of putative ABC transporter systems (PG0280, PG0281, PG1175, PG1663, PG2199 and PG2206). PG1175 and PG1663 are each predicted to be the inner membrane components of an ABC transporter complex, each having an N-terminal transmembrane domain and a C-terminal ABC ATPase domain. Interestingly, a RPSBLAST search based on the conserved domain database CDD [57] revealed that PG0280 and PG0281 encode putative permeases belonging to the family which includes LolC that has been shown to transport lipids across the inner membrane [58].

### Potential virulence determinants and hypothetical genes

The complete *P. gingivalis *genome sequence has revealed a number of putative virulence determinants, several of which were highly up-regulated in biofilm cells. These include a putative sialidase (PG0352) and ADP-heptose-LPS heptosyltransferase (PG1155) with an average fold change of 3.22 and 2.58 respectively, a putative extracellular protease (PG0553) and thiol protease, *tpr *(PG1055) [59] with average fold changes of 6.22 and 12.28 respectively. We also observed an increased expression of the gene encoding HtrA, a putative periplasmic serine protease (*htrA*; PG0593) with an average fold change of 2.96. HtrA is known to play a role in biofilm formation of *Streptococcus mutans *[60] and virulence in a variety of bacterial species [61-63]. In *P. gingivalis*, HtrA has been shown to confer protection against oxidative stress and be involved in long term adaptation to elevated temperature [64,65]. HtrA has also been implicated in the modulation of the activity of the gingipain cysteine proteinases at elevated temperature but it is not essential for the maturation or activation of the gingipains under normal conditions [64]. Interestingly *htrA *occurs in a predicted operon upstream of *rpoD*. In *Salmonella enterica *serovar Typhimurium [66,67] and *Yersinia enterocolitica *[68,69] an alternative sigma factor RpoE has been implicated in the regulation of *htrA *and resistance to oxidative stress. Taken together, these results suggest that perhaps HtrA in concert with RpoD may be part of a stress response that is activated during *P. gingvalis *biofilm growth.

The majority of the differentially regulated *P. gingivalis *genes were of unknown or poorly characterized function. Three of the genes encoding the hypothetical proteins, PG0914, PG0844, and PG1630 were also amongst the most highly up-regulated genes in biofilm cells with an average fold change of 11.69, 9.35 and 8.21 respectively. RPSBLAST search indicated that some of the hypothetical *P. gingivalis *proteins do have similarities to proteins of known function such as HslJ, a heat shock protein (PG0706) and DegQ, a trypsin-like serine proteases (PG0840) (Table 2).

**Table 2:** Putative functions of selected genes annotated as hypothetical that were up-regulated in *P. gingivalis *W50 biofilm cells

ORF	Putative gene product description and function*
PG0039	COG0845; AcrA, Membrane-fusion protein; Cell envelope biogenesis, outer membrane
PG0706	COG3187; HslJ, Heat shock protein; Posttranslational modification, protein turnover, chaperones
PG0840	COG0265; DegQ, Trypsin-like serine proteases, typically periplasmic, containing C-terminal PDZ domain; Posttranslational modification, protein turnover, chaperones
PG1012	COG0621; MiaB, 2-methylthioadenine synthetase; Translation, ribosomal structure and biogenesis
PG1100	COG2971; N-acetylglucosamine kinase; Carbohydrate transport and metabolism
PG2139	COG1399; Metal-binding, possibly nucleic acid-binding protein; General function prediction only

* Putative gene description and function were determined using RPSBLAST.

Comparison of our microarray results with the cell envelope proteome analysis of *P. gingivalis *W50 biofilm and planktonic cells performed by Ang et al. [15], using the same cells as in this study, indicates that 5 out of the 47 proteins that were of differential abundance in that study correlate with the protein abundances (up or down-regulated) that could be expected based on our microarray data. While this correlation is modest, it is important to bear in mind that protein cellular distribution, stability, post-translation modifications and/or turnover may result in measured protein abundances that differ from those expected from the transcriptomic data [70-72]. Some *P. gingivalis *proteins known to be associated with the outer membrane and virulence of the bacterium, such as the gingipains (RgpA and Kgp), HagA and CPG70, that were of differential abundance in the proteome study of Ang et al. [15] were not shown to be differentially expressed at the transcript level in this study. One of these proteins, the Lys-specific gingipain proteinase Kgp (PG1844) has been shown to be a major virulence factor for *P. gingivalis *in assimilating the essential nutrient haem [7]. In this current study the Kgp transcript level was unchanged between planktonic and biofilm growth. However, in the Ang et al. [15] study significantly less of the Kgp protein was found on the cell surface in the biofilm relative to planktonic cells. Kgp, along with other surface proteins, is known to be released from the cell surface by as yet undefined mechanisms to be present in the extracellular environment. Hence the transcriptomic and proteomic data from the same cells suggests that a major virulence factor, Kgp, may be released from the surface of the biofilm cells with no reduction in expression. This mobilization of a major virulence factor involved in assimilation of an essential nutrient may be an important survival mechanism for *P. gingivalis *in a biofilm.

It must be noted that the study presented here is of *P. gingivalis *grown as a monospecies biofilm and not as part of a multispecies biofilm as in subgingival dental plaque. Nonetheless the study does provide useful insights into the global events occurring when the bacterium is grown as a biofilm for an extended period, reflective of the chronic infection of the host. Analyses of *P. gingivalis *gene expression when it is grown as part of a multispecies biofilm are currently underway in our laboratory.

## Conclusion

In this study, we have shown 18% of the *P. gingivalis *W50 genome exhibited altered expression upon mature biofilm growth. Despite the intrinsic spatial physiological heterogeneity of biofilm cells we were able to identify a large subset of genes that were consistently differentially regulated within our biofilm replicates. From the downturn in transcription of genes involved in cell envelope biogenesis, DNA replication, energy production and biosynthesis of cofactors, prosthetic groups and carriers, the transcriptomic profiling indicated a biofilm phenotype of slow growth rate and reduced metabolic activity. The altered gene expression profiles observed in this study reflect the adaptive response of *P. gingivalis *to survive in a mature biofilm.

## Authors' contributions

CAS, SGD, NS and ECR designed the study. AWL performed the array and real time PCR analyses and wrote the initial draft of the manuscript. JPL carried out the continuous culture of *P. gingivalis *planktonic and biofilm cells. CAS, JB, SGD, NS and ECR revised the draft critically for important intellectual content. All authors have read and approved the manuscript.

## Supplementary Material

Additional file 1 **Genes differentially expressed in both *P. gingivalis *biofilm biological replicates arranged by functional category. **The data provided represent the genes differentially expressed in *P. gingivalis *strain W50 biofilm grown cells relative to planktonic cells, arranged in order of predicted functional role of the gene product. Click here for file

Additional file 2 **Genes differentially expressed in both *P. gingivalis *biofilm biological replicates arranged by ORF number.** The data provided represent the genes differentially expressed in *P. gingivalis *strain W50 biofilm grown cells relative to planktonic cells, arranged in order of TIGR ORF annotation. Click here for file
