# Supplementary material for: Comparative transcriptomic analysis of Porphyromonas gingivalis biofilm and planktonic cells
Source: BMC Microbiol. 2009 Jan 29;9:18. doi: 10.1186/1471-2180-9-18 (PMC2637884; doi:10.1186/1471-2180-9-18)
Supplement: Additional file 1 — Genes differentially expressed in both P. gingivalis biofilm biological replicates arranged by functional category. The data provided represent the genes differentially expressed in P. gingivalis strain W50 biofilm grown cells relative to planktonic cells, arranged in order of predicted functional role of the gene product. [file 1471-2180-9-18-S1.doc]

**Table S1 -** Genes consistently differentially expressed in both *P. gingivalis* biofilm biological replicates arranged by functional category

| *P.gingivalis* W50 genes up-regulated in both biofilm biological replicates arranged by functional category | | | | | | | |
| --- | --- | --- | --- | --- | --- | --- | --- |
| Gene ORF | Name | Gene description | Cellular role | Fold Change | | | |
|  |  |  |  | 1st biological replicate | | 2nd biological replicate | |
|  |  |  |  | Expression value (Log2) | CV | Expression value (Log2) | CV |
| PG1314 | aroC | chorismate synthase | Amino acid biosynthesis | 2.24 | 0.30 | 2.15 | 0.21 |
| PG0202 |  | uroporphyrinogen-III synthase HemD, putative | Biosynthesis of cofactors, prosthetic groups, and carriers | 1.13 | 0.08 | 1.14 | 0.20 |
| PG0531 | nadE | glutamine-dependent NAD+ synthetase |  | 1.94 | 0.09 | 1.22 | 0.09 |
| PG0599 | ribBA | 3,4-dihydroxy-2-butanone 4-phosphate synthase/GTP cyclohydrolase II |  | 0.90 | 0.57 | 1.01 | 0.32 |
| PG0678 |  | pyrazinamidase/nicotinamidase, putative |  | 0.98 | 0.15 | 1.14 | 0.09 |
| PG0924 |  | lipoprotein OlpA |  | 1.46 | 0.31 | 1.23 | 0.15 |
| PG0176 |  | cell surface protein, interruption-N | Cell envelope | 1.67 | 0.11 | 1.58 | 0.07 |
| PG0178 |  | cell surface protein, interruption-C |  | 2.07 | 0.16 | 1.95 | 0.20 |
| PG0192 | ompH-1 | cationic outer membrane protein OmpH |  | 1.78 | 0.19 | 1.51 | 0.22 |
| PG0193 | ompH-2 | cationic outer membrane protein OmpH |  | 1.86 | 0.39 | 1.62 | 0.31 |
| PG0352 |  | sialidase, putative |  | 1.89 | 0.31 | 1.45 | 0.31 |
| PG0726 |  | lipoprotein, putative |  | 1.41 | 0.21 | 1.80 | 0.10 |
| PG0821 |  | lipoprotein, putative |  | 0.98 | 0.59 | 1.51 | 0.33 |
| PG0922 |  | membrane protein, putative |  | 1.29 | 0.20 | 1.48 | 0.11 |
| PG1019 |  | lipoprotein, putative |  | 1.49 | 0.33 | 1.48 | 0.32 |
| PG1155 |  | ADP-heptose--LPS heptosyltransferase, putative |  | 1.38 | 0.23 | 1.35 | 0.07 |
| PG1711 |  | alpha-1,2-mannosidase family protein |  | 1.04 | 0.13 | 1.07 | 0.18 |
| PG1828 |  | lipoprotein, putative |  | 1.78 | 0.20 | 3.20 | 0.16 |
| PG1868 |  | membrane protein, putative |  | 1.30 | 0.30 | 1.08 | 0.34 |
| PG1950 |  | membrane protein |  | 1.13 | 0.47 | 1.00 | 0.41 |
| PG0266 |  | transposase, ISPg1-related, truncation | Disrupted reading frame | 1.88 | 0.16 | 2.03 | 0.20 |
| PG0299 |  | ISPg3, transposase, truncation |  | 1.79 | 0.10 | 1.30 | 0.17 |
| PG0828 |  | rteC protein, truncation |  | 1.39 | 0.48 | 1.44 | 0.23 |
| PG0931 |  | DNA-binding protein, histone-like family, degenerate |  | 1.61 | 0.17 | 1.82 | 0.12 |
| PG1187 |  | ISPg2, transposase, degenerate |  | 1.05 | 0.47 | 0.91 | 0.33 |
| PG1674 |  | hemagglutinin protein HagB, degenerate |  | 1.50 | 0.33 | 1.60 | 0.13 |
| PG0157 | recX | regulatory protein RecX | DNA metabolism | 1.47 | 0.10 | 1.03 | 0.10 |
| PG0384 |  | MutS2 family protein |  | 1.20 | 0.62 | 0.90 | 0.26 |
| PG1038 |  | ATP-dependent DNA helicase PcrA, putative |  | 1.26 | 0.17 | 1.02 | 0.13 |
| PG1495 | topB-2 | DNA topoisomerase III |  | 1.97 | 0.22 | 1.66 | 0.22 |
| PG1497 |  | DNA-binding protein, histone-like family |  | 2.60 | 0.09 | 2.32 | 0.07 |
| PG2040 |  | DNA-binding protein, histone-like family |  | 0.88 | 0.48 | 1.15 | 0.22 |
| PG0195 |  | rubrerythrin | Energy metabolism | 2.23 | 0.06 | 1.76 | 0.35 |
| PG0230 |  | transaldolase TalC, putative |  | 1.14 | 0.09 | 1.02 | 0.29 |
| PG1134 | trxB | thioredoxin reductase |  | 1.28 | 0.23 | 1.42 | 0.13 |
| PG1421 |  | ferredoxin, 4Fe-4S |  | 1.04 | 0.71 | 2.43 | 0.43 |
| PG1513 |  | phosphoribosyltransferase, putative/phosphoglycerate mutase family protein |  | 1.70 | 0.41 | 2.77 | 0.29 |
| PG1858 |  | flavodoxin |  | 1.44 | 0.24 | 1.23 | 0.21 |
| PG1956 | abfT-2 | 4-hydroxybutyrate CoA-transferase |  | 1.87 | 0.23 | 2.01 | 0.15 |
| PG0009 |  | ISPg5, transposase Orf1 | Mobile and extrachromosomal element functions | 0.88 | 0.26 | 0.97 | 0.16 |
| PG0019 |  | ISPg4, transposase |  | 1.06 | 0.13 | 1.22 | 0.18 |
| PG0177 |  | ISPg4, transposase |  | 1.38 | 0.19 | 1.80 | 0.12 |
| PG0194 |  | ISPg3, transposase |  | 2.24 | 0.04 | 1.88 | 0.07 |
| PG0838 |  | integrase |  | 2.22 | 0.06 | 1.59 | 0.08 |
| PG0872 |  | mobilizable transposon, xis protein |  | 1.53 | 0.14 | 1.73 | 0.19 |
| PG1032 |  | ISPg3, transposase |  | 1.98 | 0.13 | 1.51 | 0.13 |
| PG1061 |  | ISPg6, transposase |  | 0.93 | 0.44 | 1.24 | 0.32 |
| PG0196 |  | peptidase, M16 family | Protein fate | 1.07 | 0.19 | 1.29 | 0.11 |
| PG0553 |  | extracellular protease, putative |  | 2.79 | 0.18 | 2.46 | 0.14 |
| PG0593 | htrA | HtrA protein |  | 1.67 | 0.17 | 1.44 | 0.22 |
| PG1055 | tpr | thiol protease |  | 2.80 | 0.19 | 4.14 | 0.04 |
| PG1315 | slyD | peptidyl-prolyl cis-trans isomerase SlyD, FKBP-type |  | 1.64 | 0.15 | 1.34 | 0.29 |
| PG1548 |  | thiol protease/hemagglutinin PrtT precursor, authentic frameshift |  | 1.57 | 0.29 | 1.47 | 0.28 |
| PG1654 |  | D-alanyl-D-alanine dipeptidase |  | 1.16 | 0.21 | 1.08 | 0.24 |
| PG2201 | def | polypeptide deformylase |  | 2.11 | 0.20 | 1.94 | 0.24 |
| PG0037 | rplS | ribosomal protein L19 | Protein synthesis | 1.49 | 0.43 | 2.28 | 0.43 |
| PG0385 | rpsU | ribosomal protein S21 |  | 1.88 | 0.36 | 2.60 | 0.29 |
| PG0386 |  | site-specific recombinase, phage integrase family / ribosomal subunit interface protein |  | 1.93 | 0.37 | 2.59 | 0.36 |
| PG0568 | efp-1 | translation elongation factor P |  | 1.10 | 0.73 | 1.27 | 0.64 |
| PG0592 | rpmE | ribosomal protein L31 |  | 2.13 | 0.23 | 2.54 | 0.19 |
| PG0635 | prmA | ribosomal protein L11 methyltransferase |  | 1.22 | 0.20 | 1.19 | 0.12 |
| PG0796 | leuS | leucyl-tRNA synthetase |  | 1.56 | 0.19 | 1.24 | 0.22 |
| PG0969 |  | S-adenosylmethionine:tRNA ribosyltransferase-isomerase, putative |  | 1.65 | 0.19 | 1.67 | 0.14 |
| PG1012 |  | tRNA-i(6)A37 modification enzyme MiaB |  | 0.99 | 0.41 | 1.08 | 0.24 |
| PG1144 |  | peptide chain release factor 2, authentic frameshift |  | 1.28 | 0.44 | 1.15 | 0.67 |
| PG1723 | rpsT | ribosomal protein S20 |  | 1.40 | 0.26 | 2.28 | 0.16 |
| PG2140 | rpmF | ribosomal protein L32 |  | 1.94 | 0.24 | 2.46 | 0.27 |
| PG0925 | tmk | thymidine kinase | Purines, pyrimidines, nucleosides, and nucleotides | 1.68 | 0.09 | 1.08 | 0.11 |
| PG0173 |  | transcriptional regulator, putative | Regulatory functions | 1.57 | 0.14 | 1.49 | 0.22 |
| PG0826 |  | transcriptional regulator, AraC family |  | 1.05 | 0.30 | 0.92 | 0.33 |
| PG2186 |  | transcriptional regulator, putative |  | 1.68 | 0.22 | 0.95 | 0.29 |
| PG1431 |  | DNA-binding response regulator, LuxR family | Signal transduction | 1.85 | 0.33 | 2.24 | 0.29 |
| PG1432 |  | sensor histidine kinase |  | 2.56 | 0.24 | 2.18 | 0.24 |
| PG0594 | rpoD | RNA polymerase sigma-70 factor | Transcription | 1.26 | 0.17 | 1.44 | 0.10 |
| PG0923 | rbfA | ribosome-binding factor A |  | 1.27 | 0.23 | 1.00 | 0.14 |
| PG1660 |  | RNA polymerase sigma-70 factor, ECF subfamily |  | 0.91 | 0.20 | 1.48 | 0.08 |
| PG1688 | greA | transcription elongation factor GreA |  | 1.98 | 0.35 | 1.90 | 0.33 |
| PG0063 |  | outer membrane efflux protein | Transport and binding proteins | 1.11 | 0.33 | 1.52 | 0.18 |
| PG0064 |  | heavy metal efflux pump, CzcA family |  | 1.29 | 0.34 | 1.86 | 0.16 |
| PG0065 |  | efflux transporter, RND family, MFP subunit |  | 1.31 | 0.46 | 1.99 | 0.09 |
| PG0280 |  | ABC transporter, permease protein, putative |  | 0.94 | 0.42 | 1.30 | 0.31 |
| PG0281 |  | ABC transporter, permease protein, putative |  | 1.03 | 0.51 | 1.35 | 0.20 |
| PG0283 |  | efflux transporter, RND family, MFP subunit |  | 1.03 | 0.38 | 0.92 | 0.49 |
| PG1101 |  | sodium:solute symporter family protein |  | 1.89 | 0.10 | 1.19 | 0.09 |
| PG1175 |  | ABC transporter, ATP-binding protein, putative |  | 1.29 | 0.43 | 0.94 | 0.44 |
| PG1383 |  | amino acid exporter, putative |  | 1.21 | 0.31 | 1.27 | 0.21 |
| PG1446 |  | MATE efflux family protein |  | 1.09 | 0.33 | 1.05 | 0.19 |
| PG1663 |  | ABC transporter, ATP-binding protein |  | 1.44 | 0.29 | 0.94 | 0.06 |
| PG1666 |  | efflux transporter, MFP component, RND family |  | 0.89 | 0.27 | 1.06 | 0.19 |
| PG1667 |  | outer membrane efflux protein |  | 1.04 | 0.35 | 1.06 | 0.12 |
| PG2185 |  | transporter, putative |  | 1.84 | 0.12 | 1.50 | 0.18 |
| PG2199 |  | ABC transporter, ATP-binding protein, putative |  | 2.53 | 0.14 | 2.29 | 0.16 |
| PG2206 |  | ABC transporter, ATP-binding protein |  | 1.11 | 0.52 | 0.93 | 0.32 |
| PG0158 |  | competence protein F-related protein | Unknown function | 2.33 | 0.12 | 1.75 | 0.10 |
| PG0174 |  | pyridine nucleotide-disulphide oxidoreductase family protein |  | 1.56 | 0.27 | 1.63 | 0.19 |
| PG0199 |  | TatD family protein |  | 0.94 | 0.40 | 1.12 | 0.18 |
| PG0449 |  | TPR domain protein |  | 1.32 | 0.41 | 0.95 | 0.39 |
| PG0587 | yadS | yadS protein |  | 1.19 | 0.43 | 1.52 | 0.17 |
| PG0666 |  | mdsC protein, authentic frameshift |  | 0.88 | 0.90 | 0.97 | 0.73 |
| PG0769 |  | fibronectin type III domain protein |  | 2.19 | 0.09 | 1.31 | 0.29 |
| PG1099 |  | glucokinase regulator-related protein |  | 1.47 | 0.13 | 1.09 | 0.11 |
| PG1156 |  | S4 domain protein |  | 1.49 | 0.09 | 1.55 | 0.06 |
| PG1174 |  | thioesterase family protein |  | 1.53 | 0.25 | 1.69 | 0.07 |
| PG1235 |  | epimerase/reductase, putative |  | 1.00 | 0.27 | 1.50 | 0.20 |
| PG1374 |  | immunoreactive 47 kDa antigen PG97 |  | 1.12 | 0.12 | 1.87 | 0.08 |
| PG1514 |  | glycerol dehydrogenase-related protein |  | 1.77 | 0.25 | 2.15 | 0.07 |
| PG1515 |  | ribulose bisphosphate carboxylase-related protein |  | 1.15 | 0.33 | 1.28 | 0.36 |
| PG1570 |  | rhodanese-like domain protein |  | 1.47 | 0.16 | 1.08 | 0.19 |
| PG1579 |  | ATPase, MoxR family |  | 2.11 | 0.15 | 1.52 | 0.22 |
| PG1687 |  | HIT family protein |  | 1.76 | 0.30 | 1.97 | 0.19 |
| PG2028 |  | ebsC protein |  | 0.94 | 0.15 | 0.98 | 0.14 |
| PG2100 |  | immunoreactive 63 kDa antigen PG102 |  | 1.32 | 0.44 | 3.22 | 0.05 |
| PG2102 |  | immunoreactive 61 kDa antigen PG91 |  | 0.86 | 0.80 | 3.54 | 0.10 |
| PG2200 |  | TPR domain protein |  | 1.53 | 0.15 | 1.11 | 0.05 |
| PG0018 |  | hypothetical protein | Hypothetical protein | 1.05 | 0.37 | 0.83 | 0.27 |
| PG0039 |  | hypothetical protein |  | 1.45 | 0.08 | 2.12 | 0.16 |
| PG0066 |  | hypothetical protein |  | 0.99 | 0.35 | 1.44 | 0.16 |
| PG0197 |  | hypothetical protein |  | 0.92 | 0.45 | 0.90 | 0.24 |
| PG0229 |  | hypothetical protein |  | 1.94 | 0.21 | 2.09 | 0.28 |
| PG0250 |  | hypothetical protein |  | 0.98 | 0.32 | 1.11 | 0.22 |
| PG0265 |  | hypothetical protein |  | 2.02 | 0.18 | 1.67 | 0.24 |
| PG0313 |  | hypothetical protein |  | 1.00 | 0.43 | 0.91 | 0.27 |
| PG0339 |  | hypothetical protein |  | 1.34 | 0.19 | 1.19 | 0.20 |
| PG0340 |  | hypothetical protein |  | 1.21 | 0.18 | 0.84 | 0.22 |
| PG0351 |  | hypothetical protein |  | 2.22 | 0.23 | 1.79 | 0.17 |
| PG0354 |  | hypothetical protein |  | 1.66 | 0.58 | 0.91 | 0.26 |
| PG0409 |  | hypothetical protein |  | 2.64 | 0.09 | 1.51 | 0.05 |
| PG0419 |  | hypothetical protein |  | 3.01 | 0.16 | 2.17 | 0.27 |
| PG0457 |  | hypothetical protein |  | 1.17 | 0.08 | 1.47 | 0.07 |
| PG0494 |  | hypothetical protein |  | 1.24 | 0.16 | 1.56 | 0.14 |
| PG0536 |  | hypothetical protein |  | 2.20 | 0.23 | 2.26 | 0.06 |
| PG0556 |  | hypothetical protein |  | 1.01 | 0.51 | 1.22 | 0.27 |
| PG0610 |  | hypothetical protein |  | 1.25 | 0.66 | 1.12 | 0.74 |
| PG0614 |  | hypothetical protein |  | 1.83 | 0.23 | 0.86 | 0.29 |
| PG0681 |  | hypothetical protein |  | 2.07 | 0.34 | 1.79 | 0.36 |
| PG0706 |  | hypothetical protein |  | 1.05 | 0.67 | 0.82 | 0.69 |
| PG0722 |  | hypothetical protein |  | 1.50 | 0.19 | 1.24 | 0.23 |
| PG0749 |  | hypothetical protein |  | 1.33 | 0.30 | 1.53 | 0.21 |
| PG0770 |  | hypothetical protein |  | 1.74 | 0.12 | 1.13 | 0.14 |
| PG0840 |  | hypothetical protein |  | 1.73 | 0.17 | 1.10 | 0.31 |
| PG0844 |  | hypothetical protein |  | 3.73 | 0.17 | 2.44 | 0.15 |
| PG0848 |  | hypothetical protein |  | 1.35 | 0.14 | 1.03 | 0.22 |
| PG0856 |  | hypothetical protein |  | 0.96 | 0.61 | 1.14 | 0.75 |
| PG0871 |  | hypothetical protein |  | 1.18 | 0.34 | 1.77 | 0.26 |
| PG0914 |  | hypothetical protein |  | 3.90 | 0.15 | 3.08 | 0.13 |
| PG0994 |  | hypothetical protein |  | 1.59 | 0.49 | 1.29 | 0.40 |
| PG0995 |  | hypothetical protein |  | 1.91 | 0.13 | 1.52 | 0.15 |
| PG1021 |  | hypothetical protein |  | 0.97 | 0.45 | 1.14 | 0.24 |
| PG1059 |  | hypothetical protein |  | 1.78 | 0.29 | 1.17 | 0.30 |
| PG1085 |  | hypothetical protein |  | 0.88 | 0.44 | 1.26 | 0.80 |
| PG1100 |  | hypothetical protein |  | 1.65 | 0.18 | 1.11 | 0.23 |
| PG1102 |  | hypothetical protein |  | 2.64 | 0.09 | 1.48 | 0.06 |
| PG1108 |  | hypothetical protein |  | 1.28 | 0.23 | 1.25 | 0.58 |
| PG1196 |  | hypothetical protein |  | 1.02 | 0.15 | 0.85 | 0.34 |
| PG1222 |  | hypothetical protein |  | 1.04 | 0.37 | 1.60 | 0.25 |
| PG1250 |  | hypothetical protein |  | 1.22 | 0.15 | 1.39 | 0.24 |
| PG1251 |  | hypothetical protein |  | 1.03 | 0.22 | 1.18 | 0.11 |
| PG1304 |  | hypothetical protein |  | 2.56 | 0.12 | 2.09 | 0.12 |
| PG1316 |  | hypothetical protein |  | 1.74 | 0.22 | 1.40 | 0.25 |
| PG1317 |  | hypothetical protein |  | 2.03 | 0.20 | 1.61 | 0.07 |
| PG1363 |  | conserved domain protein |  | 1.41 | 0.15 | 1.46 | 0.15 |
| PG1488 |  | hypothetical protein |  | 1.19 | 0.35 | 1.40 | 0.31 |
| PG1496 |  | hypothetical protein |  | 0.91 | 0.36 | 1.42 | 0.18 |
| PG1508 |  | hypothetical protein |  | 2.43 | 0.13 | 0.84 | 0.58 |
| PG1549 |  | hypothetical protein |  | 1.30 | 0.52 | 0.95 | 0.55 |
| PG1626 |  | hypothetical protein |  | 1.22 | 0.37 | 0.86 | 0.24 |
| PG1630 |  | hypothetical protein |  | 3.27 | 0.08 | 2.76 | 0.10 |
| PG1634 |  | hypothetical protein |  | 2.06 | 0.31 | 2.11 | 0.26 |
| PG1635 |  | hypothetical protein |  | 1.80 | 0.24 | 1.61 | 0.11 |
| PG1659 |  | hypothetical protein |  | 1.10 | 0.23 | 1.43 | 0.17 |
| PG1662 |  | hypothetical protein |  | 1.12 | 0.22 | 1.11 | 0.14 |
| PG1715 |  | hypothetical protein |  | 2.35 | 0.19 | 1.77 | 0.19 |
| PG1722 |  | hypothetical protein |  | 1.00 | 0.40 | 0.96 | 0.35 |
| PG1786 |  | hypothetical protein |  | 1.54 | 0.12 | 2.00 | 0.05 |
| PG1795 |  | hypothetical protein |  | 0.98 | 0.21 | 1.15 | 0.21 |
| PG1908 |  | hypothetical protein |  | 1.34 | 0.36 | 1.18 | 0.58 |
| PG1969 |  | hypothetical protein |  | 1.38 | 0.10 | 1.84 | 0.07 |
| PG1970 |  | hypothetical protein |  | 1.46 | 0.34 | 0.83 | 0.32 |
| PG1974 |  | hypothetical protein |  | 1.04 | 0.19 | 1.47 | 0.10 |
| PG1977 |  | hypothetical protein |  | 1.61 | 0.22 | 1.29 | 0.15 |
| PG2029 |  | hypothetical protein |  | 1.06 | 0.28 | 1.29 | 0.12 |
| PG2136 |  | hypothetical protein |  | 1.49 | 0.19 | 0.91 | 0.24 |
| PG2139 |  | hypothetical protein |  | 1.51 | 0.04 | 2.49 | 0.14 |
| PG2216 |  | hypothetical protein |  | 1.52 | 0.46 | 0.96 | 0.36 |
| PG2220 |  | hypothetical protein |  | 0.92 | 0.27 | 1.10 | 0.19 |
| PG0231 |  | conserved hypothetical protein |  | 1.70 | 0.22 | 0.99 | 0.20 |
| PG0686 |  | conserved hypothetical protein |  | 1.35 | 0.47 | 1.03 | 0.58 |
| PG1489 |  | conserved hypothetical protein |  | 1.21 | 0.35 | 0.91 | 0.36 |
| PG1494 |  | hypothetical protein |  | 2.25 | 0.14 | 2.31 | 0.05 |
| PG1754 |  | conserved domain protein |  | 1.44 | 0.21 | 1.05 | 0.13 |
| PG1874 |  | conserved hypothetical protein |  | 1.19 | 0.20 | 1.14 | 0.06 |

| *P.gingivalis* W50 genes down-regulated in both biofilm biological replicates arranged by functional category | | | |  |  |  |  |
| --- | --- | --- | --- | --- | --- | --- | --- |
| Gene ID | Name | Gene description | Cellular role | Fold Change | |  |  |
|  |  |  |  | 1st biological replicate | | 2nd biological replicate | |
|  |  |  |  | Expression value (Log2) | CV | Expression value (Log2) | CV |
| PG0885 |  | phospho-2-dehydro-3-deoxyheptonate aldolase/chorismate mutase | Amino acid biosynthesis | -1.30 | -0.26 | -1.77 | -0.15 |
| PG0210 |  | precorrin-6x reductase/cobalamin biosynthetic protein CbiD | Biosynthesis of cofactors, prosthetic groups, and carriers | -0.95 | -0.56 | -0.87 | -0.59 |
| PG0211 | cbiGF | cobalamin biosynthesis protein CbiG, putative/precorrin-4 methyltransferase |  | -1.12 | -0.41 | -0.93 | -0.42 |
| PG0212 | cobL | precorrin-6y c5,15-methyltransferase, putative |  | -0.99 | -0.45 | -1.14 | -0.25 |
| PG0446 | thiF | thiF protein |  | -1.37 | -0.48 | -1.36 | -0.32 |
| PG0475 |  | oxygen-independent coproporphyrinogen III oxidase, putative |  | -2.17 | -0.22 | -1.39 | -0.53 |
| PG0480 |  | precorrin-2 C20-methyltransferase, putative |  | -1.37 | -0.21 | -1.25 | -0.65 |
| PG0957 | ribF | riboflavin biosynthesis protein RibF |  | -1.15 | -0.20 | -0.92 | -0.25 |
| PG1521 |  | O-succinylbenzoic acid--CoA ligase |  | -1.00 | -0.45 | -1.30 | -0.15 |
| PG1577 | nadC | nicotinate-nucleotide pyrophosphorylase |  | -1.02 | -0.20 | -0.93 | -0.09 |
| PG1578 | nadA | quinolinate synthetase complex, subunit A |  | -0.92 | -0.15 | -1.02 | -0.18 |
| PG1619 |  | biotin synthesis protein BioC, putative |  | -0.88 | -0.60 | -0.89 | -0.53 |
| PG1851 | coaBC | dfp protein |  | -1.20 | -0.12 | -1.09 | -0.10 |
| PG2061 | folA | dihydrofolate reductase |  | -1.14 | -0.08 | -1.29 | -0.23 |
| PG2159 |  | protoporphyrinogen oxidase |  | -2.25 | -0.17 | -2.20 | -0.30 |
| PG0670 |  | lipoprotein, putative | Cell envelope | -1.56 | -0.27 | -1.48 | -0.26 |
| PG0698 |  | lipoprotein, putative |  | -0.87 | -0.40 | -1.05 | -0.11 |
| PG1097 |  | Mur ligase domain protein/alanine racemase |  | -1.71 | -0.21 | -1.43 | -0.20 |
| PG1140 |  | glycosyl transferase, group 2 family protein |  | -1.22 | -0.15 | -0.92 | -0.15 |
| PG1141 |  | glycosyl transferase, group 1 family protein |  | -1.22 | -0.34 | -0.79 | -0.24 |
| PG1342 | murB | UDP-N-acetylenolpyruvoylglucosamine reductase |  | -1.49 | -0.21 | -1.55 | -0.04 |
| PG1422 | dacB | D-alanyl-D-alanine carboxypeptidase |  | -1.28 | -0.51 | -1.93 | -0.31 |
| PG1560 | rfbB | dTDP-glucose 4,6-dehydratase |  | -1.23 | -0.14 | -0.83 | -0.34 |
| PG1561 | rfbD | dTDP-4-dehydrorhamnose reductase |  | -1.39 | -0.04 | -1.30 | -0.15 |
| PG1562 | rfbC | dTDP-4-dehydrorhamnose 3,5-epimerase |  | -1.19 | -0.08 | -0.98 | -0.14 |
| PG1600 |  | membrane protein, putative |  | -2.30 | -0.38 | -2.30 | -0.46 |
| PG1880 |  | glycosyl transferase, group 2 family protein |  | -1.45 | -0.37 | -1.73 | -0.27 |
| PG2095 |  | lipoprotein, putative |  | -0.92 | -0.40 | -0.83 | -0.34 |
| PG2133 |  | lipoprotein, putative |  | -1.14 | -0.29 | -1.34 | -0.51 |
| PG2134 |  | lipoprotein, putative |  | -0.99 | -0.35 | -1.11 | -0.25 |
| PG2173 | omp28 | outer membrane lipoprotein Omp28 |  | -0.93 | -0.22 | -0.88 | -0.12 |
| PG2223 |  | glycosyl transferase, group 2 family protein |  | -1.15 | -0.48 | -0.89 | -0.49 |
| PG0618 |  | alkyl hydroperoxide reductase, C subunit | Cellular processes | -1.68 | -0.10 | -1.20 | -0.09 |
| PG0619 |  | alkyl hydroperoxide reductase, F subunit |  | -2.29 | -0.15 | -1.11 | -0.20 |
| PG1648 |  | RelA/SpoT family protein |  | -1.05 | -0.50 | -1.30 | -0.26 |
| PG2163 | surE | stationary-phase survival protein SurE |  | -1.66 | -0.09 | -0.92 | -0.27 |
| PG1075 |  | coenzyme A transferase, beta subunit | Central intermediary metabolism | -1.09 | -0.23 | -1.38 | -0.07 |
| PG0001 | dnaA | chromosomal replication initiator protein DnaA | DNA metabolism | -1.69 | -0.09 | -1.10 | -0.11 |
| PG0035 | dnaE | DNA polymerase III, alpha subunit |  | -1.14 | -0.27 | -0.91 | -0.28 |
| PG0227 | radA | DNA repair protein RadA |  | -0.86 | -0.37 | -1.33 | -0.25 |
| PG0271 | ssb | single-stranded binding protein |  | -1.14 | -0.28 | -1.00 | -0.24 |
| PG0412 | mutL | DNA mismatch repair protein MutL |  | -1.00 | -0.47 | -1.04 | -0.40 |
| PG0971 |  | McrBC restriction endonuclease system, McrB subunit, putative |  | -1.26 | -0.46 | -1.64 | -0.20 |
| PG1072 |  | MutS family protein |  | -0.98 | -0.15 | -1.20 | -0.19 |
| PG1469 |  | type I restriction-modification system, M subunit, putative |  | -1.32 | -0.74 | -0.87 | -0.80 |
| PG1696 |  | type II DNA modification methyltransferase, putative |  | -1.21 | -0.42 | -1.17 | -0.18 |
| PG1697 |  | type II restriction endonuclease, putative |  | -1.57 | -0.33 | -1.50 | -0.08 |
| PG1735 |  | MutT/nudix family protein |  | -0.99 | -0.36 | -0.82 | -0.40 |
| PG1849 | recN | DNA repair protein RecN |  | -1.23 | -0.21 | -1.27 | -0.21 |
| PG1853 | dnaN | DNA polymerase III, beta subunit |  | -0.96 | -0.31 | -0.85 | -0.23 |
| PG1993 | uvrC | excinuclease ABC, C subunit |  | -0.92 | -0.85 | -1.19 | -1.21 |
| PG2032 | priA | primosomal protein n' |  | -0.86 | -1.65 | -1.19 | -1.21 |
| PG0474 |  | low-specificity L-threonine aldolase, putative | Energy metabolism | -1.37 | -0.26 | -0.86 | -0.59 |
| PG0675 | iorA | indolepyruvate ferredoxin oxidoreductase, alpha subunit |  | -1.09 | -0.25 | -1.16 | -0.16 |
| PG0690 | abfT-1 | 4-hydroxybutyrate CoA-transferase |  | -0.95 | -0.13 | -0.94 | -0.15 |
| PG0699 | malP | maltodextrin phosphorylase |  | -1.05 | -0.25 | -1.23 | -0.09 |
| PG1042 |  | glycogen synthase, putative |  | -1.03 | -0.96 | -1.46 | -0.73 |
| PG1073 | kamD | D-lysine 5,6-aminomutase, alpha subunit |  | -1.08 | -0.18 | -1.16 | -0.14 |
| PG1077 | etfB-2 | electron transfer flavoprotein, beta subunit |  | -1.34 | -0.21 | -1.33 | -0.10 |
| PG1608 | mmdB | methylmalonyl-CoA decarboxylase, beta subunit |  | -1.08 | -0.63 | -0.97 | -0.60 |
| PG1609 | mmdC | methylmalonyl-CoA decarboxylase, gamma subunit |  | -1.16 | -0.25 | -1.09 | -0.14 |
| PG1612 | mmdA | methylmalonyl-CoA decarboxylase, alpha subunit |  | -1.15 | -0.27 | -1.16 | -0.26 |
| PG1676 | pckA | phosphoenolpyruvate carboxykinase (ATP) |  | -1.74 | -0.14 | -0.84 | -0.39 |
| PG1747 |  | ribose 5-phosphate isomerase B, putative |  | -1.30 | -0.12 | -1.30 | -0.12 |
| PG1812 |  | 2-oxoglutarate oxidoreductase, alpha subunit |  | -1.22 | -0.16 | -0.98 | -0.20 |
| PG2145 |  | polysaccharide deacetylase |  | -1.59 | -0.20 | -1.35 | -0.43 |
| PG1076 | acdA | acyl-CoA dehydrogenase, short-chain specific | Fatty acid and phospholipid metabolism | -1.68 | -0.16 | -1.69 | -0.08 |
| PG1079 |  | enoyl-CoA hydratase/isomerase family protein |  | -1.16 | -0.13 | -1.27 | -0.16 |
| PG1983 |  | CRISPR-associated protein, TM1791 family | Mobile and extrachromosomal element functions | -0.83 | -0.51 | -0.93 | -0.20 |
| PG1984 |  | CRISPR-associated protein, TM1791.1 family |  | -0.85 | -0.47 | -1.17 | -0.15 |
| PG1985 |  | CRISPR-associated protein, TM1792 family |  | -1.34 | -0.33 | -1.62 | -0.12 |
| PG1986 |  | CRISPR-associated protein, Cmr3 family (cmr3) |  | -1.19 | -0.15 | -1.76 | -0.11 |
| PG1987 |  | CRISPR-associated protein, TM1794 family |  | -1.09 | -0.51 | -1.22 | -0.33 |
| PG2015 | cas4 | CRISPR-associated protein, Cas4 |  | -1.34 | -0.49 | -1.22 | -0.32 |
| PG2016 | cas3 | CRISPR-associated protein, Cas3 |  | -1.18 | -0.58 | -0.97 | -0.48 |
| PG2020 |  | CRISPR-associated protein, TM1814 family |  | -1.01 | -0.26 | -0.95 | -0.20 |
| PG0521 | groES | chaperonin, 10 kDa | Protein fate | -1.46 | -0.68 | -1.73 | -0.49 |
| PG0956 |  | peptidase, M23/M37 family, putative |  | -1.49 | -0.17 | -1.21 | -0.11 |
| PG1210 |  | peptidase, M24 family protein |  | -0.88 | -0.40 | -0.82 | -0.17 |
| PG1343 | lipB | lipoate-protein ligase B |  | -1.60 | -0.12 | -1.47 | -0.12 |
| PG1598 |  | lipoprotein signal peptidase, putative |  | -1.07 | -0.53 | -0.97 | -0.42 |
| PG1601 |  | biotin--acetyl-CoA-carboxylase ligase |  | -1.12 | -0.42 | -1.29 | -0.15 |
| PG2088 | msrA | peptide methionine sulfoxide reductase |  | -1.45 | -0.08 | -1.26 | -0.14 |
| PG0099 | pheT | phenylalanyl-tRNA synthetase, beta subunit | Protein synthesis | -1.02 | -0.52 | -0.80 | -0.39 |
| PG0135 | ksgA | dimethyladenosine transferase |  | -0.98 | -0.26 | -1.06 | -0.35 |
| PG0248 |  | translation initation factor SUI1, putative |  | -0.87 | -0.62 | -0.91 | -0.31 |
| PG0522 | miaA-2 | tRNA delta(2)-isopentenylpyrophosphate transferase |  | -1.48 | -0.21 | -1.28 | -0.14 |
| PG0755 |  | tRNA pseudouridine synthase A |  | -1.22 | -0.18 | -0.80 | -0.22 |
| PG1095 |  | RNA methyltransferase, TrmA family |  | -1.18 | -0.17 | -0.84 | -0.36 |
| PG1878 | cysS | cysteinyl-tRNA synthetase |  | -1.00 | -0.39 | -0.90 | -0.20 |
| PG1994 | dtd | D-tyrosyl-tRNA(Tyr) deacylase |  | -1.13 | -0.59 | -1.35 | -0.27 |
| PG0512 | gmk | guanylate kinase | Purines, pyrimidines, nucleosides, and nucleotides | -1.56 | -0.12 | -1.74 | -0.05 |
| PG0270 | oxyR | redox-sensitive transcriptional activator OxyR | Regulatory functions | -1.00 | -0.44 | -1.05 | -0.31 |
| PG1138 | porR | pigmentation and extracellular proteinase regulator |  | -1.46 | -0.33 | -1.05 | -0.31 |
| PG1797 |  | DNA-binding response regulator/sensor histidine kinase | Signal transduction | -1.10 | -0.41 | -0.83 | -0.26 |
| PG1213 | rnhA | ribonuclease H | Transcription | -1.56 | -0.07 | -1.54 | -0.10 |
| PG1827 |  | RNA polymerase sigma-70 factor, ECF subfamily |  | -1.35 | -0.42 | -0.84 | -1.23 |
| PG0644 |  | TonB-linked receptor Tlr, authentic frameshift | Transport and binding proteins | -1.13 | -0.28 | -1.18 | -0.13 |
| PG0671 |  | iron compound ABC transporter, permease protein |  | -1.00 | -0.65 | -1.16 | -0.36 |
| PG0672 |  | iron compound ABC transporter, ATP-binding protein |  | -1.18 | -0.28 | -1.10 | -0.15 |
| PG0912 |  | polysaccharide transport protein, putative |  | -1.07 | -0.33 | -1.07 | -0.44 |
| PG0938 |  | calcium-transporting ATPase |  | -0.93 | -0.43 | -1.31 | -0.98 |
| PG1126 | uraA | uracil permease |  | -0.93 | -0.87 | -1.11 | -0.36 |
| PG1176 |  | ABC transporter, ATP-binding protein, putative |  | -1.27 | -0.30 | -1.16 | -0.22 |
| PG1294 | feoB-2 | ferrous iron transport protein B |  | -1.05 | -0.48 | -0.90 | -0.30 |
| PG1734 |  | transporter, putative |  | -1.17 | -0.55 | -1.27 | -0.47 |
| PG1965 |  | voltage gated chloride channel, authentic frameshift |  | -1.02 | -0.83 | -1.26 | -0.45 |
| PG0061 | yngK-1 | yngK protein | Unknown function | -1.27 | -0.23 | -1.00 | -0.30 |
| PG0062 |  | TPR domain protein |  | -1.65 | -0.19 | -1.31 | -0.20 |
| PG0240 |  | hydrolase, haloacid dehalogenase-like family |  | -1.26 | -0.42 | -0.81 | -0.59 |
| PG0272 |  | CBS domain protein |  | -1.38 | -0.17 | -1.02 | -0.31 |
| PG0509 |  | prenyltransferase, UbiA family |  | -1.58 | -0.41 | -1.31 | -0.41 |
| PG0511 |  | spore maturation protein A/spore maturation protein B |  | -1.36 | -0.50 | -1.59 | -0.32 |
| PG0691 |  | NifU-related protein |  | -1.20 | -0.07 | -1.51 | -0.08 |
| PG0954 |  | TPR domain protein |  | -0.84 | -0.34 | -0.95 | -0.11 |
| PG1137 | porS | porS protein |  | -3.01 | -0.31 | -1.01 | -1.02 |
| PG1142 |  | exopolysaccharide synthesis-related protein |  | -1.02 | -0.64 | -1.31 | -0.45 |
| PG1211 |  | hexapeptide transferase family protein |  | -1.15 | -0.24 | -1.27 | -0.11 |
| PG1212 |  | TPR domain protein |  | -0.97 | -0.32 | -1.18 | -0.16 |
| PG1404 |  | rhomboid family protein |  | -1.07 | -0.48 | -1.02 | -0.35 |
| PG1594 |  | ComEC/Rec2-related protein |  | -1.44 | -0.57 | -0.99 | -0.70 |
| PG1727 | yitL | yitL protein |  | -1.09 | -0.19 | -0.97 | -0.12 |
| PG1728 |  | cytidine/deoxycytidylate deaminase family protein |  | -1.21 | -0.27 | -0.81 | -0.21 |
| PG1842 |  | acetyltransferase, GNAT family |  | -1.17 | -0.49 | -0.85 | -0.77 |
| PG1852 |  | exonuclease |  | -0.85 | -0.11 | -0.79 | -0.14 |
| PG1864 |  | leucine-rich protein |  | -1.21 | -0.28 | -1.16 | -0.15 |
| PG2047 |  | helicase, putative |  | -0.85 | -0.50 | -1.03 | -0.46 |
| PG2072 |  | UvrD/REP helicase domain protein |  | -0.98 | -0.38 | -1.13 | -0.14 |
| PG0028 | ispF | conserved hypothetical protein TIGR00151 | Hypothetical protein | -1.14 | -0.44 | -0.90 | -0.52 |
| PG0055 |  | conserved domain protein |  | -0.83 | -0.82 | -1.30 | -0.30 |
| PG0059 |  | hypothetical protein |  | -1.18 | -0.20 | -0.98 | -0.48 |
| PG0060 |  | hypothetical protein |  | -1.49 | -0.34 | -1.59 | -0.43 |
| PG0287 |  | hypothetical protein |  | -1.02 | -0.26 | -0.80 | -0.25 |
| PG0325 |  | conserved hypothetical protein |  | -1.73 | -0.15 | -0.98 | -0.26 |
| PG0327 |  | hypothetical protein |  | -2.02 | -0.29 | -1.23 | -0.72 |
| PG0447 |  | conserved hypothetical protein |  | -1.12 | -0.18 | -1.23 | -0.19 |
| PG0510 |  | conserved hypothetical protein |  | -1.52 | -0.13 | -1.29 | -0.12 |
| PG0513 |  | conserved hypothetical protein TIGR00255 |  | -1.37 | -0.35 | -1.44 | -0.17 |
| PG0547 |  | conserved hypothetical protein |  | -1.47 | -0.20 | -1.01 | -0.21 |
| PG0774 |  | hypothetical protein |  | -1.08 | -0.53 | -0.94 | -0.44 |
| PG0780 |  | hypothetical protein |  | -0.88 | -0.18 | -1.05 | -0.13 |
| PG0781 |  | hypothetical protein |  | -0.84 | -0.29 | -0.93 | -0.31 |
| PG0884 |  | hypothetical protein |  | -1.30 | -0.27 | -1.56 | -0.12 |
| PG0886 |  | hypothetical protein |  | -1.29 | -0.60 | -1.39 | -0.40 |
| PG0888 |  | hypothetical protein |  | -1.17 | -0.15 | -0.88 | -0.19 |
| PG0901 |  | conserved hypothetical protein |  | -1.46 | -0.19 | -1.55 | -0.14 |
| PG0955 |  | hypothetical protein |  | -0.84 | -0.18 | -1.02 | -0.28 |
| PG0972 |  | conserved hypothetical protein |  | -0.97 | -0.43 | -1.89 | -0.47 |
| PG1071 |  | hypothetical protein |  | -1.06 | -0.16 | -1.09 | -0.09 |
| PG1125 |  | hypothetical protein |  | -1.29 | -0.51 | -1.25 | -0.29 |
| PG1136 |  | hypothetical protein |  | -2.52 | -0.39 | -1.26 | -0.47 |
| PG1206 |  | hypothetical protein |  | -1.64 | -0.11 | -1.14 | -0.22 |
| PG1207 |  | hypothetical protein |  | -1.69 | -0.41 | -1.07 | -0.27 |
| PG1209 |  | hypothetical protein |  | -0.85 | -0.17 | -1.14 | -0.07 |
| PG1282 |  | conserved hypothetical protein |  | -1.18 | -0.33 | -1.51 | -0.23 |
| PG1351 |  | hypothetical protein |  | -1.00 | -0.20 | -1.22 | -0.11 |
| PG1359 |  | hypothetical protein |  | -1.49 | -0.64 | -1.52 | -0.27 |
| PG1528 |  | conserved hypothetical protein |  | -1.14 | -0.53 | -1.02 | -0.51 |
| PG1588 |  | conserved hypothetical protein |  | -1.28 | -0.16 | -1.20 | -0.37 |
| PG1610 |  | hypothetical protein |  | -1.43 | -0.10 | -1.54 | -0.06 |
| PG1611 |  | hypothetical protein |  | -1.63 | -0.10 | -1.59 | -0.12 |
| PG1618 |  | conserved hypothetical protein |  | -1.01 | -0.54 | -0.82 | -0.47 |
| PG1733 |  | hypothetical protein |  | -1.34 | -0.30 | -1.48 | -0.22 |
| PG1738 |  | hypothetical protein |  | -1.48 | -0.18 | -0.95 | -0.32 |
| PG1791 |  | hypothetical protein |  | -1.07 | -0.42 | -0.89 | -0.31 |
| PG1850 |  | hypothetical protein |  | -1.18 | -0.28 | -1.33 | -0.23 |
| PG1857 |  | hypothetical protein |  | -2.79 | -0.23 | -1.20 | -0.79 |
| PG1861 |  | hypothetical protein |  | -0.99 | -0.31 | -1.51 | -0.25 |
| PG1862 |  | hypothetical protein |  | -1.05 | -0.23 | -1.11 | -0.31 |
| PG1863 |  | hypothetical protein |  | -1.00 | -0.27 | -0.92 | -0.13 |
| PG1871 |  | hypothetical protein |  | -1.06 | -0.36 | -0.83 | -0.28 |
| PG1879 |  | conserved hypothetical protein |  | -1.16 | -0.30 | -1.05 | -0.12 |
| PG1881 |  | hypothetical protein |  | -1.17 | -0.35 | -0.97 | -0.10 |
| PG1888 |  | conserved hypothetical protein |  | -1.02 | -0.44 | -0.81 | -0.19 |
| PG1892 |  | hypothetical protein |  | -0.86 | -0.49 | -0.86 | -0.36 |
| PG1895 |  | hypothetical protein |  | -1.63 | -0.37 | -1.14 | -0.65 |
| PG1900 |  | conserved hypothetical protein |  | -0.92 | -0.14 | -1.16 | -0.33 |
| PG1988 |  | hypothetical protein |  | -1.50 | -0.53 | -1.49 | -0.40 |
| PG1989 |  | hypothetical protein |  | -1.72 | -0.41 | -2.01 | -0.22 |
| PG1995 |  | conserved hypothetical protein |  | -1.12 | -0.54 | -1.37 | -0.28 |
| PG2017 |  | hypothetical protein |  | -1.79 | -0.51 | -1.78 | -0.39 |
| PG2018 |  | hypothetical protein |  | -1.07 | -0.65 | -1.72 | -0.31 |
| PG2019 |  | hypothetical protein |  | -1.13 | -0.24 | -1.52 | -0.09 |
| PG2087 |  | conserved hypothetical protein |  | -1.54 | -0.31 | -1.41 | -0.26 |
| PG2089 |  | hypothetical protein |  | -1.27 | -0.30 | -1.11 | -0.31 |
| PG2096 |  | conserved domain protein |  | -0.86 | -0.40 | -0.89 | -0.18 |
