# Supplementary material for: Comparative transcriptomic analysis of Porphyromonas gingivalis biofilm and planktonic cells
Source: BMC Microbiol. 2009 Jan 29;9:18. doi: 10.1186/1471-2180-9-18 (PMC2637884; doi:10.1186/1471-2180-9-18)
Supplement: Additional file 2 — Genes differentially expressed in both P. gingivalis biofilm biological replicates arranged by ORF number. The data provided represent the genes differentially expressed in P. gingivalis strain W50 biofilm grown cells relative to planktonic cells, arranged in order of TIGR ORF annotation. [file 1471-2180-9-18-S2.doc]

| Table S2. Genes consistently differentially expressed in both *P. gingivalis* biofilm biological replicates arranged by ORF number | | | |  |  |  |  |
| --- | --- | --- | --- | --- | --- | --- | --- |
| *P.gingivalis* W50 genes up-regulated in both biofilm biological replicates arranged by ORF number | | | |  |  |  |  |
| ORF | Name | Gene description | Cellular role | Fold Change | |  |  |
|  |  |  |  | 1st biological replicate | | 2nd biological replicate | |
|  |  |  |  | Expression value (Log2) | CV | Expression value (Log2) | CV |
| PG0009 |  | ISPg5, transposase Orf1 | Mobile and extrachromosomal element functions | 0.88 | 0.26 | 0.97 | 0.16 |
| PG0018 |  | hypothetical protein | Hypothetical protein | 1.05 | 0.37 | 0.83 | 0.27 |
| PG0019 |  | ISPg4, transposase | Mobile and extrachromosomal element functions | 1.06 | 0.13 | 1.22 | 0.18 |
| PG0037 | rplS | ribosomal protein L19 | Protein synthesis | 1.49 | 0.43 | 2.28 | 0.43 |
| PG0039 |  | hypothetical protein | Hypothetical protein | 1.45 | 0.08 | 2.12 | 0.16 |
| PG0063 |  | outer membrane efflux protein | Transport and binding proteins | 1.11 | 0.33 | 1.52 | 0.18 |
| PG0064 |  | heavy metal efflux pump, CzcA family | Transport and binding proteins | 1.29 | 0.34 | 1.86 | 0.16 |
| PG0065 |  | efflux transporter, RND family, MFP subunit | Transport and binding proteins | 1.31 | 0.46 | 1.99 | 0.09 |
| PG0066 |  | hypothetical protein | Hypothetical protein | 0.99 | 0.35 | 1.44 | 0.16 |
| PG0157 | recX | regulatory protein RecX | DNA metabolism | 1.47 | 0.10 | 1.03 | 0.10 |
| PG0158 |  | competence protein F-related protein | Unknown function | 2.33 | 0.12 | 1.75 | 0.10 |
| PG0173 |  | transcriptional regulator, putative | Regulatory functions | 1.57 | 0.14 | 1.49 | 0.22 |
| PG0174 |  | pyridine nucleotide-disulphide oxidoreductase family protein | Unknown function | 1.56 | 0.27 | 1.63 | 0.19 |
| PG0176 |  | cell surface protein, interruption-N | Cell envelope | 1.67 | 0.11 | 1.58 | 0.07 |
| PG0177 |  | ISPg4, transposase | Mobile and extrachromosomal element functions | 1.38 | 0.19 | 1.80 | 0.12 |
| PG0178 |  | cell surface protein, interruption-C | Cell envelope | 2.07 | 0.16 | 1.95 | 0.20 |
| PG0192 | ompH-1 | cationic outer membrane protein OmpH | Cell envelope | 1.78 | 0.19 | 1.51 | 0.22 |
| PG0193 | ompH-2 | cationic outer membrane protein OmpH | Cell envelope | 1.86 | 0.39 | 1.62 | 0.31 |
| PG0194 |  | ISPg3, transposase | Mobile and extrachromosomal element functions | 2.24 | 0.04 | 1.88 | 0.07 |
| PG0195 |  | rubrerythrin | Energy metabolism | 2.23 | 0.06 | 1.76 | 0.35 |
| PG0196 |  | peptidase, M16 family | Protein fate | 1.07 | 0.19 | 1.29 | 0.11 |
| PG0197 |  | hypothetical protein | Hypothetical protein | 0.92 | 0.45 | 0.90 | 0.24 |
| PG0199 |  | TatD family protein | Unknown function | 0.94 | 0.40 | 1.12 | 0.18 |
| PG0202 |  | uroporphyrinogen-III synthase HemD, putative | Biosynthesis of cofactors, prosthetic groups, and carriers | 1.13 | 0.08 | 1.14 | 0.20 |
| PG0229 |  | hypothetical protein | Hypothetical protein | 1.94 | 0.21 | 2.09 | 0.28 |
| PG0230 |  | transaldolase TalC, putative | Energy metabolism | 1.14 | 0.09 | 1.02 | 0.29 |
| PG0231 |  | conserved hypothetical protein | Hypothetical protein | 1.70 | 0.22 | 0.99 | 0.20 |
| PG0250 |  | hypothetical protein | Hypothetical protein | 0.98 | 0.32 | 1.11 | 0.22 |
| PG0265 |  | hypothetical protein | Hypothetical protein | 2.02 | 0.18 | 1.67 | 0.24 |
| PG0266 |  | transposase, ISPg1-related, truncation | Disrupted reading frame | 1.88 | 0.16 | 2.03 | 0.20 |
| PG0280 |  | ABC transporter, permease protein, putative | Transport and binding proteins | 0.94 | 0.42 | 1.30 | 0.31 |
| PG0281 |  | ABC transporter, permease protein, putative | Transport and binding proteins | 1.03 | 0.51 | 1.35 | 0.20 |
| PG0283 |  | efflux transporter, RND family, MFP subunit | Transport and binding proteins | 1.03 | 0.38 | 0.92 | 0.49 |
| PG0299 |  | ISPg3, transposase, truncation | Disrupted reading frame | 1.79 | 0.10 | 1.30 | 0.17 |
| PG0313 |  | hypothetical protein | Hypothetical protein | 1.00 | 0.43 | 0.91 | 0.27 |
| PG0339 |  | hypothetical protein | Hypothetical protein | 1.34 | 0.19 | 1.19 | 0.20 |
| PG0340 |  | hypothetical protein | Hypothetical protein | 1.21 | 0.18 | 0.84 | 0.22 |
| PG0351 |  | hypothetical protein | Hypothetical protein | 2.22 | 0.23 | 1.79 | 0.17 |
| PG0352 |  | sialidase, putative | Cell envelope | 1.89 | 0.31 | 1.45 | 0.31 |
| PG0354 |  | hypothetical protein | Hypothetical protein | 1.66 | 0.58 | 0.91 | 0.26 |
| PG0384 |  | MutS2 family protein | DNA metabolism | 1.20 | 0.62 | 0.90 | 0.26 |
| PG0385 | rpsU | ribosomal protein S21 | Protein synthesis | 1.88 | 0.36 | 2.60 | 0.29 |
| PG0386 |  | site-specific recombinase, phage integrase family / ribosomal subunit interface protein | Protein synthesis | 1.93 | 0.37 | 2.59 | 0.36 |
| PG0409 |  | hypothetical protein | Hypothetical protein | 2.64 | 0.09 | 1.51 | 0.05 |
| PG0419 |  | hypothetical protein | Hypothetical protein | 3.01 | 0.16 | 2.17 | 0.27 |
| PG0449 |  | TPR domain protein | Unknown function | 1.32 | 0.41 | 0.95 | 0.39 |
| PG0457 |  | hypothetical protein | Hypothetical protein | 1.17 | 0.08 | 1.47 | 0.07 |
| PG0494 |  | hypothetical protein | Hypothetical protein | 1.24 | 0.16 | 1.56 | 0.14 |
| PG0531 | nadE | glutamine-dependent NAD+ synthetase | Biosynthesis of cofactors, prosthetic groups, and carriers | 1.94 | 0.09 | 1.22 | 0.09 |
| PG0536 |  | hypothetical protein | Hypothetical protein | 2.20 | 0.23 | 2.26 | 0.06 |
| PG0553 |  | extracellular protease, putative | Protein fate | 2.79 | 0.18 | 2.46 | 0.14 |
| PG0556 |  | hypothetical protein | Hypothetical protein | 1.01 | 0.51 | 1.22 | 0.27 |
| PG0568 | efp-1 | translation elongation factor P | Protein synthesis | 1.10 | 0.73 | 1.27 | 0.64 |
| PG0587 | yadS | yadS protein | Unknown function | 1.19 | 0.43 | 1.52 | 0.17 |
| PG0592 | rpmE | ribosomal protein L31 | Protein synthesis | 2.13 | 0.23 | 2.54 | 0.19 |
| PG0593 | htrA | HtrA protein | Protein fate | 1.67 | 0.17 | 1.44 | 0.22 |
| PG0594 | rpoD | RNA polymerase sigma-70 factor | Transcription | 1.26 | 0.17 | 1.44 | 0.10 |
| PG0599 | ribBA | 3,4-dihydroxy-2-butanone 4-phosphate synthase/GTP cyclohydrolase II | Biosynthesis of cofactors, prosthetic groups, and carriers | 0.90 | 0.57 | 1.01 | 0.32 |
| PG0610 |  | hypothetical protein | Hypothetical protein | 1.25 | 0.66 | 1.12 | 0.74 |
| PG0614 |  | hypothetical protein | Hypothetical protein | 1.83 | 0.23 | 0.86 | 0.29 |
| PG0635 | prmA | ribosomal protein L11 methyltransferase | Protein synthesis | 1.22 | 0.20 | 1.19 | 0.12 |
| PG0666 |  | mdsC protein, authentic frameshift | Unknown function | 0.88 | 0.90 | 0.97 | 0.73 |
| PG0678 |  | pyrazinamidase/nicotinamidase, putative | Biosynthesis of cofactors, prosthetic groups, and carriers | 0.98 | 0.15 | 1.14 | 0.09 |
| PG0681 |  | hypothetical protein | Hypothetical protein | 2.07 | 0.34 | 1.79 | 0.36 |
| PG0686 |  | conserved hypothetical protein | Hypothetical protein | 1.35 | 0.47 | 1.03 | 0.58 |
| PG0706 |  | hypothetical protein | Hypothetical protein | 1.05 | 0.67 | 0.82 | 0.69 |
| PG0722 |  | hypothetical protein | Hypothetical protein | 1.50 | 0.19 | 1.24 | 0.23 |
| PG0726 |  | lipoprotein, putative | Cell envelope | 1.41 | 0.21 | 1.80 | 0.10 |
| PG0749 |  | hypothetical protein | Hypothetical protein | 1.33 | 0.30 | 1.53 | 0.21 |
| PG0769 |  | fibronectin type III domain protein | Unknown function | 2.19 | 0.09 | 1.31 | 0.29 |
| PG0770 |  | hypothetical protein | Hypothetical protein | 1.74 | 0.12 | 1.13 | 0.14 |
| PG0796 | leuS | leucyl-tRNA synthetase | Protein synthesis | 1.56 | 0.19 | 1.24 | 0.22 |
| PG0821 |  | lipoprotein, putative | Cell envelope | 0.98 | 0.59 | 1.51 | 0.33 |
| PG0826 |  | transcriptional regulator, AraC family | Regulatory functions | 1.05 | 0.30 | 0.92 | 0.33 |
| PG0828 |  | rteC protein, truncation | Disrupted reading frame | 1.39 | 0.48 | 1.44 | 0.23 |
| PG0838 |  | integrase | Mobile and extrachromosomal element functions | 2.22 | 0.06 | 1.59 | 0.08 |
| PG0840 |  | hypothetical protein | Hypothetical protein | 1.73 | 0.17 | 1.10 | 0.31 |
| PG0844 |  | hypothetical protein | Hypothetical protein | 3.73 | 0.17 | 2.44 | 0.15 |
| PG0848 |  | hypothetical protein | Hypothetical protein | 1.35 | 0.14 | 1.03 | 0.22 |
| PG0856 |  | hypothetical protein | Hypothetical protein | 0.96 | 0.61 | 1.14 | 0.75 |
| PG0871 |  | hypothetical protein | Hypothetical protein | 1.18 | 0.34 | 1.77 | 0.26 |
| PG0872 |  | mobilizable transposon, xis protein | Mobile and extrachromosomal element functions | 1.53 | 0.14 | 1.73 | 0.19 |
| PG0914 |  | hypothetical protein | Hypothetical protein | 3.90 | 0.15 | 3.08 | 0.13 |
| PG0922 |  | membrane protein, putative | Cell envelope | 1.29 | 0.20 | 1.48 | 0.11 |
| PG0923 | rbfA | ribosome-binding factor A | Transcription | 1.27 | 0.23 | 1.00 | 0.14 |
| PG0924 |  | lipoprotein OlpA | Biosynthesis of cofactors, prosthetic groups, and carriers | 1.46 | 0.31 | 1.23 | 0.15 |
| PG0925 | tmk | thymidine kinase | Purines, pyrimidines, nucleosides, and nucleotides | 1.68 | 0.09 | 1.08 | 0.11 |
| PG0931 |  | DNA-binding protein, histone-like family, degenerate | Disrupted reading frame | 1.61 | 0.17 | 1.82 | 0.12 |
| PG0969 |  | S-adenosylmethionine:tRNA ribosyltransferase-isomerase, putative | Protein synthesis | 1.65 | 0.19 | 1.67 | 0.14 |
| PG0994 |  | hypothetical protein | Hypothetical protein | 1.59 | 0.49 | 1.29 | 0.40 |
| PG0995 |  | hypothetical protein | Hypothetical protein | 1.91 | 0.13 | 1.52 | 0.15 |
| PG1012 |  | tRNA-i(6)A37 modification enzyme MiaB | Protein synthesis | 0.99 | 0.41 | 1.08 | 0.24 |
| PG1019 |  | lipoprotein, putative | Cell envelope | 1.49 | 0.33 | 1.48 | 0.32 |
| PG1021 |  | hypothetical protein | Hypothetical protein | 0.97 | 0.45 | 1.14 | 0.24 |
| PG1032 |  | ISPg3, transposase | Mobile and extrachromosomal element functions | 1.98 | 0.13 | 1.51 | 0.13 |
| PG1038 |  | ATP-dependent DNA helicase PcrA, putative | DNA metabolism | 1.26 | 0.17 | 1.02 | 0.13 |
| PG1055 | tpr | thiol protease | Protein fate | 2.80 | 0.19 | 4.14 | 0.04 |
| PG1059 |  | hypothetical protein | Hypothetical protein | 1.78 | 0.29 | 1.17 | 0.30 |
| PG1061 |  | ISPg6, transposase | Mobile and extrachromosomal element functions | 0.93 | 0.44 | 1.24 | 0.32 |
| PG1085 |  | hypothetical protein | Hypothetical protein | 0.88 | 0.44 | 1.26 | 0.80 |
| PG1099 |  | glucokinase regulator-related protein | Unknown function | 1.47 | 0.13 | 1.09 | 0.11 |
| PG1100 |  | hypothetical protein | Hypothetical protein | 1.65 | 0.18 | 1.11 | 0.23 |
| PG1101 |  | sodium:solute symporter family protein | Transport and binding proteins | 1.89 | 0.10 | 1.19 | 0.09 |
| PG1102 |  | hypothetical protein | Hypothetical protein | 2.64 | 0.09 | 1.48 | 0.06 |
| PG1108 |  | hypothetical protein | Hypothetical protein | 1.28 | 0.23 | 1.25 | 0.58 |
| PG1134 | trxB | thioredoxin reductase | Energy metabolism | 1.28 | 0.23 | 1.42 | 0.13 |
| PG1144 |  | peptide chain release factor 2, authentic frameshift | Protein synthesis | 1.28 | 0.44 | 1.15 | 0.67 |
| PG1155 |  | ADP-heptose--LPS heptosyltransferase, putative | Cell envelope | 1.38 | 0.23 | 1.35 | 0.07 |
| PG1156 |  | S4 domain protein | Unknown function | 1.49 | 0.09 | 1.55 | 0.06 |
| PG1174 |  | thioesterase family protein | Unknown function | 1.53 | 0.25 | 1.69 | 0.07 |
| PG1175 |  | ABC transporter, ATP-binding protein, putative | Transport and binding proteins | 1.29 | 0.43 | 0.94 | 0.44 |
| PG1187 |  | ISPg2, transposase, degenerate | Disrupted reading frame | 1.05 | 0.47 | 0.91 | 0.33 |
| PG1196 |  | hypothetical protein | Hypothetical protein | 1.02 | 0.15 | 0.85 | 0.34 |
| PG1222 |  | hypothetical protein | Hypothetical protein | 1.04 | 0.37 | 1.60 | 0.25 |
| PG1235 |  | epimerase/reductase, putative | Unknown function | 1.00 | 0.27 | 1.50 | 0.20 |
| PG1250 |  | hypothetical protein | Hypothetical protein | 1.22 | 0.15 | 1.39 | 0.24 |
| PG1251 |  | hypothetical protein | Hypothetical protein | 1.03 | 0.22 | 1.18 | 0.11 |
| PG1304 |  | hypothetical protein | Hypothetical protein | 2.56 | 0.12 | 2.09 | 0.12 |
| PG1314 | aroC | chorismate synthase | Amino acid biosynthesis | 2.24 | 0.30 | 2.15 | 0.21 |
| PG1315 | slyD | peptidyl-prolyl cis-trans isomerase SlyD, FKBP-type | Protein fate | 1.64 | 0.15 | 1.34 | 0.29 |
| PG1316 |  | hypothetical protein | Hypothetical protein | 1.74 | 0.22 | 1.40 | 0.25 |
| PG1317 |  | hypothetical protein | Hypothetical protein | 2.03 | 0.20 | 1.61 | 0.07 |
| PG1363 |  | conserved domain protein | Hypothetical protein | 1.41 | 0.15 | 1.46 | 0.15 |
| PG1374 |  | immunoreactive 47 kDa antigen PG97 | Unknown function | 1.12 | 0.12 | 1.87 | 0.08 |
| PG1383 |  | amino acid exporter, putative | Transport and binding proteins | 1.21 | 0.31 | 1.27 | 0.21 |
| PG1421 |  | ferredoxin, 4Fe-4S | Energy metabolism | 1.04 | 0.71 | 2.43 | 0.43 |
| PG1431 |  | DNA-binding response regulator, LuxR family | Signal transduction | 1.85 | 0.33 | 2.24 | 0.29 |
| PG1432 |  | sensor histidine kinase | Signal transduction | 2.56 | 0.24 | 2.18 | 0.24 |
| PG1446 |  | MATE efflux family protein | Transport and binding proteins | 1.09 | 0.33 | 1.05 | 0.19 |
| PG1488 |  | hypothetical protein | Hypothetical protein | 1.19 | 0.35 | 1.40 | 0.31 |
| PG1489 |  | conserved hypothetical protein | Hypothetical protein | 1.21 | 0.35 | 0.91 | 0.36 |
| PG1494 |  | hypothetical protein | Hypothetical protein | 2.25 | 0.14 | 2.31 | 0.05 |
| PG1495 | topB-2 | DNA topoisomerase III | DNA metabolism | 1.97 | 0.22 | 1.66 | 0.22 |
| PG1496 |  | hypothetical protein | Hypothetical protein | 0.91 | 0.36 | 1.42 | 0.18 |
| PG1497 |  | DNA-binding protein, histone-like family | DNA metabolism | 2.60 | 0.09 | 2.32 | 0.07 |
| PG1508 |  | hypothetical protein | Hypothetical protein | 2.43 | 0.13 | 0.84 | 0.58 |
| PG1513 |  | phosphoribosyltransferase, putative/phosphoglycerate mutase family protein | Energy metabolism | 1.70 | 0.41 | 2.77 | 0.29 |
| PG1514 |  | glycerol dehydrogenase-related protein | Unknown function | 1.77 | 0.25 | 2.15 | 0.07 |
| PG1515 |  | ribulose bisphosphate carboxylase-related protein | Unknown function | 1.15 | 0.33 | 1.28 | 0.36 |
| PG1548 |  | thiol protease/hemagglutinin PrtT precursor, authentic frameshift | Protein fate | 1.57 | 0.29 | 1.47 | 0.28 |
| PG1549 |  | hypothetical protein | Hypothetical protein | 1.30 | 0.52 | 0.95 | 0.55 |
| PG1570 |  | rhodanese-like domain protein | Unknown function | 1.47 | 0.16 | 1.08 | 0.19 |
| PG1579 |  | ATPase, MoxR family | Unknown function | 2.11 | 0.15 | 1.52 | 0.22 |
| PG1626 |  | hypothetical protein | Hypothetical protein | 1.22 | 0.37 | 0.86 | 0.24 |
| PG1630 |  | hypothetical protein | Hypothetical protein | 3.27 | 0.08 | 2.76 | 0.10 |
| PG1634 |  | hypothetical protein | Hypothetical protein | 2.06 | 0.31 | 2.11 | 0.26 |
| PG1635 |  | hypothetical protein | Hypothetical protein | 1.80 | 0.24 | 1.61 | 0.11 |
| PG1654 |  | D-alanyl-D-alanine dipeptidase | Protein fate | 1.16 | 0.21 | 1.08 | 0.24 |
| PG1659 |  | hypothetical protein | Hypothetical protein | 1.10 | 0.23 | 1.43 | 0.17 |
| PG1660 |  | RNA polymerase sigma-70 factor, ECF subfamily | Transcription | 0.91 | 0.20 | 1.48 | 0.08 |
| PG1662 |  | hypothetical protein | Hypothetical protein | 1.12 | 0.22 | 1.11 | 0.14 |
| PG1663 |  | ABC transporter, ATP-binding protein | Transport and binding proteins | 1.44 | 0.29 | 0.94 | 0.06 |
| PG1666 |  | efflux transporter, MFP component, RND family | Transport and binding proteins | 0.89 | 0.27 | 1.06 | 0.19 |
| PG1667 |  | outer membrane efflux protein | Transport and binding proteins | 1.04 | 0.35 | 1.06 | 0.12 |
| PG1674 |  | hemagglutinin protein HagB, degenerate | Disrupted reading frame | 1.50 | 0.33 | 1.60 | 0.13 |
| PG1687 |  | HIT family protein | Unknown function | 1.76 | 0.30 | 1.97 | 0.19 |
| PG1688 | greA | transcription elongation factor GreA | Transcription | 1.98 | 0.35 | 1.90 | 0.33 |
| PG1711 |  | alpha-1,2-mannosidase family protein | Cell envelope | 1.04 | 0.13 | 1.07 | 0.18 |
| PG1715 |  | hypothetical protein | Hypothetical protein | 2.35 | 0.19 | 1.77 | 0.19 |
| PG1722 |  | hypothetical protein | Hypothetical protein | 1.00 | 0.40 | 0.96 | 0.35 |
| PG1723 | rpsT | ribosomal protein S20 | Protein synthesis | 1.40 | 0.26 | 2.28 | 0.16 |
| PG1754 |  | conserved domain protein | Hypothetical protein | 1.44 | 0.21 | 1.05 | 0.13 |
| PG1786 |  | hypothetical protein | Hypothetical protein | 1.54 | 0.12 | 2.00 | 0.05 |
| PG1795 |  | hypothetical protein | Hypothetical protein | 0.98 | 0.21 | 1.15 | 0.21 |
| PG1828 |  | lipoprotein, putative | Cell envelope | 1.78 | 0.20 | 3.20 | 0.16 |
| PG1858 |  | flavodoxin | Energy metabolism | 1.44 | 0.24 | 1.23 | 0.21 |
| PG1868 |  | membrane protein, putative | Cell envelope | 1.30 | 0.30 | 1.08 | 0.34 |
| PG1874 |  | conserved hypothetical protein | Hypothetical protein | 1.19 | 0.20 | 1.14 | 0.06 |
| PG1908 |  | hypothetical protein | Hypothetical protein | 1.34 | 0.36 | 1.18 | 0.58 |
| PG1950 |  | membrane protein | Cell envelope | 1.13 | 0.47 | 1.00 | 0.41 |
| PG1956 | abfT-2 | 4-hydroxybutyrate CoA-transferase | Energy metabolism | 1.87 | 0.23 | 2.01 | 0.15 |
| PG1969 |  | hypothetical protein | Hypothetical protein | 1.38 | 0.10 | 1.84 | 0.07 |
| PG1970 |  | hypothetical protein | Hypothetical protein | 1.46 | 0.34 | 0.83 | 0.32 |
| PG1974 |  | hypothetical protein | Hypothetical protein | 1.04 | 0.19 | 1.47 | 0.10 |
| PG1977 |  | hypothetical protein | Hypothetical protein | 1.61 | 0.22 | 1.29 | 0.15 |
| PG2028 |  | ebsC protein | Unknown function | 0.94 | 0.15 | 0.98 | 0.14 |
| PG2029 |  | hypothetical protein | Hypothetical protein | 1.06 | 0.28 | 1.29 | 0.12 |
| PG2040 |  | DNA-binding protein, histone-like family | DNA metabolism | 0.88 | 0.48 | 1.15 | 0.22 |
| PG2100 |  | immunoreactive 63 kDa antigen PG102 | Unknown function | 1.32 | 0.44 | 3.22 | 0.05 |
| PG2102 |  | immunoreactive 61 kDa antigen PG91 | Unknown function | 0.86 | 0.80 | 3.54 | 0.10 |
| PG2136 |  | hypothetical protein | Hypothetical protein | 1.49 | 0.19 | 0.91 | 0.24 |
| PG2139 |  | hypothetical protein | Hypothetical protein | 1.51 | 0.04 | 2.49 | 0.14 |
| PG2140 | rpmF | ribosomal protein L32 | Protein synthesis | 1.94 | 0.24 | 2.46 | 0.27 |
| PG2185 |  | transporter, putative | Transport and binding proteins | 1.84 | 0.12 | 1.50 | 0.18 |
| PG2186 |  | transcriptional regulator, putative | Regulatory functions | 1.68 | 0.22 | 0.95 | 0.29 |
| PG2199 |  | ABC transporter, ATP-binding protein, putative | Transport and binding proteins | 2.53 | 0.14 | 2.29 | 0.16 |
| PG2200 |  | TPR domain protein | Unknown function | 1.53 | 0.15 | 1.11 | 0.05 |
| PG2201 | def | polypeptide deformylase | Protein fate | 2.11 | 0.20 | 1.94 | 0.24 |
| PG2206 |  | ABC transporter, ATP-binding protein | Transport and binding proteins | 1.11 | 0.52 | 0.93 | 0.32 |
| PG2216 |  | hypothetical protein | Hypothetical protein | 1.52 | 0.46 | 0.96 | 0.36 |
| PG2220 |  | hypothetical protein | Hypothetical protein | 0.92 | 0.27 | 1.10 | 0.19 |
|  |  |  |  |  |  |  |  |
| *P.gingivalis* W50 genes down-regulated in both biofilm biological replicates arranged by ORF number | | | |  |  |  |  |
| Gene ID | Name | Gene description | Cellular role | Fold Change | |  |  |
|  |  |  |  | 1st biological replicate | | 2nd biological replicate | |
|  |  |  |  | Expression value (Log2) | CV | Expression value (Log2) | CV |
| PG0001 | dnaA | chromosomal replication initiator protein DnaA | DNA metabolism | -1.69 | -0.09 | -1.10 | -0.11 |
| PG0028 | ispF | conserved hypothetical protein TIGR00151 | Hypothetical protein | -1.14 | -0.44 | -0.90 | -0.52 |
| PG0035 | dnaE | DNA polymerase III, alpha subunit | DNA metabolism | -1.14 | -0.27 | -0.91 | -0.28 |
| PG0055 |  | conserved domain protein | Hypothetical protein | -0.83 | -0.82 | -1.30 | -0.30 |
| PG0059 |  | hypothetical protein | Hypothetical protein | -1.18 | -0.20 | -0.98 | -0.48 |
| PG0060 |  | hypothetical protein | Hypothetical protein | -1.49 | -0.34 | -1.59 | -0.43 |
| PG0061 | yngK-1 | yngK protein | Unknown function | -1.27 | -0.23 | -1.00 | -0.30 |
| PG0062 |  | TPR domain protein | Unknown function | -1.65 | -0.19 | -1.31 | -0.20 |
| PG0099 | pheT | phenylalanyl-tRNA synthetase, beta subunit | Protein synthesis | -1.02 | -0.52 | -0.80 | -0.39 |
| PG0135 | ksgA | dimethyladenosine transferase | Protein synthesis | -0.98 | -0.26 | -1.06 | -0.35 |
| PG0210 |  | precorrin-6x reductase/cobalamin biosynthetic protein CbiD | Biosynthesis of cofactors, prosthetic groups, and carriers | -0.95 | -0.56 | -0.87 | -0.59 |
| PG0211 | cbiGF | cobalamin biosynthesis protein CbiG, putative/precorrin-4 methyltransferase | Biosynthesis of cofactors, prosthetic groups, and carriers | -1.12 | -0.41 | -0.93 | -0.42 |
| PG0212 | cobL | precorrin-6y c5,15-methyltransferase, putative | Biosynthesis of cofactors, prosthetic groups, and carriers | -0.99 | -0.45 | -1.14 | -0.25 |
| PG0227 | radA | DNA repair protein RadA | DNA metabolism | -0.86 | -0.37 | -1.33 | -0.25 |
| PG0240 |  | hydrolase, haloacid dehalogenase-like family | Unknown function | -1.26 | -0.42 | -0.81 | -0.59 |
| PG0248 |  | translation initation factor SUI1, putative | Protein synthesis | -0.87 | -0.62 | -0.91 | -0.31 |
| PG0270 | oxyR | redox-sensitive transcriptional activator OxyR | Regulatory functions | -1.00 | -0.44 | -1.05 | -0.31 |
| PG0271 | ssb | single-stranded binding protein | DNA metabolism | -1.14 | -0.28 | -1.00 | -0.24 |
| PG0272 |  | CBS domain protein | Unknown function | -1.38 | -0.17 | -1.02 | -0.31 |
| PG0287 |  | hypothetical protein | Hypothetical protein | -1.02 | -0.26 | -0.80 | -0.25 |
| PG0325 |  | conserved hypothetical protein | Hypothetical protein | -1.73 | -0.15 | -0.98 | -0.26 |
| PG0327 |  | hypothetical protein | Hypothetical protein | -2.02 | -0.29 | -1.23 | -0.72 |
| PG0412 | mutL | DNA mismatch repair protein MutL | DNA metabolism | -1.00 | -0.47 | -1.04 | -0.40 |
| PG0446 | thiF | thiF protein | Biosynthesis of cofactors, prosthetic groups, and carriers | -1.37 | -0.48 | -1.36 | -0.32 |
| PG0447 |  | conserved hypothetical protein | Hypothetical protein | -1.12 | -0.18 | -1.23 | -0.19 |
| PG0474 |  | low-specificity L-threonine aldolase, putative | Energy metabolism | -1.37 | -0.26 | -0.86 | -0.59 |
| PG0475 |  | oxygen-independent coproporphyrinogen III oxidase, putative | Biosynthesis of cofactors, prosthetic groups, and carriers | -2.17 | -0.22 | -1.39 | -0.53 |
| PG0480 |  | precorrin-2 C20-methyltransferase, putative | Biosynthesis of cofactors, prosthetic groups, and carriers | -1.37 | -0.21 | -1.25 | -0.65 |
| PG0509 |  | prenyltransferase, UbiA family | Unknown function | -1.58 | -0.41 | -1.31 | -0.41 |
| PG0510 |  | conserved hypothetical protein | Hypothetical protein | -1.52 | -0.13 | -1.29 | -0.12 |
| PG0511 |  | spore maturation protein A/spore maturation protein B | Unknown function | -1.36 | -0.50 | -1.59 | -0.32 |
| PG0512 | gmk | guanylate kinase | Purines, pyrimidines, nucleosides, and nucleotides | -1.56 | -0.12 | -1.74 | -0.05 |
| PG0513 |  | conserved hypothetical protein TIGR00255 | Hypothetical protein | -1.37 | -0.35 | -1.44 | -0.17 |
| PG0521 | groES | chaperonin, 10 kDa | Protein fate | -1.46 | -0.68 | -1.73 | -0.49 |
| PG0522 | miaA-2 | tRNA delta(2)-isopentenylpyrophosphate transferase | Protein synthesis | -1.48 | -0.21 | -1.28 | -0.14 |
| PG0547 |  | conserved hypothetical protein | Hypothetical protein | -1.47 | -0.20 | -1.01 | -0.21 |
| PG0618 |  | alkyl hydroperoxide reductase, C subunit | Cellular processes | -1.68 | -0.10 | -1.20 | -0.09 |
| PG0619 |  | alkyl hydroperoxide reductase, F subunit | Cellular processes | -2.29 | -0.15 | -1.11 | -0.20 |
| PG0644 |  | TonB-linked receptor Tlr, authentic frameshift | Transport and binding proteins | -1.13 | -0.28 | -1.18 | -0.13 |
| PG0670 |  | lipoprotein, putative | Cell envelope | -1.56 | -0.27 | -1.48 | -0.26 |
| PG0671 |  | iron compound ABC transporter, permease protein | Transport and binding proteins | -1.00 | -0.65 | -1.16 | -0.36 |
| PG0672 |  | iron compound ABC transporter, ATP-binding protein | Transport and binding proteins | -1.18 | -0.28 | -1.10 | -0.15 |
| PG0675 | iorA | indolepyruvate ferredoxin oxidoreductase, alpha subunit | Energy metabolism | -1.09 | -0.25 | -1.16 | -0.16 |
| PG0690 | abfT-1 | 4-hydroxybutyrate CoA-transferase | Energy metabolism | -0.95 | -0.13 | -0.94 | -0.15 |
| PG0691 |  | NifU-related protein | Unknown function | -1.20 | -0.07 | -1.51 | -0.08 |
| PG0698 |  | lipoprotein, putative | Cell envelope | -0.87 | -0.40 | -1.05 | -0.11 |
| PG0699 | malP | maltodextrin phosphorylase | Energy metabolism | -1.05 | -0.25 | -1.23 | -0.09 |
| PG0755 |  | tRNA pseudouridine synthase A | Protein synthesis | -1.22 | -0.18 | -0.80 | -0.22 |
| PG0774 |  | hypothetical protein | Hypothetical protein | -1.08 | -0.53 | -0.94 | -0.44 |
| PG0780 |  | hypothetical protein | Hypothetical protein | -0.88 | -0.18 | -1.05 | -0.13 |
| PG0781 |  | hypothetical protein | Hypothetical protein | -0.84 | -0.29 | -0.93 | -0.31 |
| PG0884 |  | hypothetical protein | Hypothetical protein | -1.30 | -0.27 | -1.56 | -0.12 |
| PG0885 |  | phospho-2-dehydro-3-deoxyheptonate aldolase/chorismate mutase | Amino acid biosynthesis | -1.30 | -0.26 | -1.77 | -0.15 |
| PG0886 |  | hypothetical protein | Hypothetical protein | -1.29 | -0.60 | -1.39 | -0.40 |
| PG0888 |  | hypothetical protein | Hypothetical protein | -1.17 | -0.15 | -0.88 | -0.19 |
| PG0901 |  | conserved hypothetical protein | Hypothetical protein | -1.46 | -0.19 | -1.55 | -0.14 |
| PG0912 |  | polysaccharide transport protein, putative | Transport and binding proteins | -1.07 | -0.33 | -1.07 | -0.44 |
| PG0938 |  | calcium-transporting ATPase | Transport and binding proteins | -0.93 | -0.43 | -1.31 | -0.98 |
| PG0954 |  | TPR domain protein | Unknown function | -0.84 | -0.34 | -0.95 | -0.11 |
| PG0955 |  | hypothetical protein | Hypothetical protein | -0.84 | -0.18 | -1.02 | -0.28 |
| PG0956 |  | peptidase, M23/M37 family, putative | Protein fate | -1.49 | -0.17 | -1.21 | -0.11 |
| PG0957 | ribF | riboflavin biosynthesis protein RibF | Biosynthesis of cofactors, prosthetic groups, and carriers | -1.15 | -0.20 | -0.92 | -0.25 |
| PG0971 |  | McrBC restriction endonuclease system, McrB subunit, putative | DNA metabolism | -1.26 | -0.46 | -1.64 | -0.20 |
| PG0972 |  | conserved hypothetical protein | Hypothetical protein | -0.97 | -0.43 | -1.89 | -0.47 |
| PG1042 |  | glycogen synthase, putative | Energy metabolism | -1.03 | -0.96 | -1.46 | -0.73 |
| PG1071 |  | hypothetical protein | Hypothetical protein | -1.06 | -0.16 | -1.09 | -0.09 |
| PG1072 |  | MutS family protein | DNA metabolism | -0.98 | -0.15 | -1.20 | -0.19 |
| PG1073 | kamD | D-lysine 5,6-aminomutase, alpha subunit | Energy metabolism | -1.08 | -0.18 | -1.16 | -0.14 |
| PG1075 |  | coenzyme A transferase, beta subunit | Central intermediary metabolism | -1.09 | -0.23 | -1.38 | -0.07 |
| PG1076 | acdA | acyl-CoA dehydrogenase, short-chain specific | Fatty acid and phospholipid metabolism | -1.68 | -0.16 | -1.69 | -0.08 |
| PG1077 | etfB-2 | electron transfer flavoprotein, beta subunit | Energy metabolism | -1.34 | -0.21 | -1.33 | -0.10 |
| PG1079 |  | enoyl-CoA hydratase/isomerase family protein | Fatty acid and phospholipid metabolism | -1.16 | -0.13 | -1.27 | -0.16 |
| PG1095 |  | RNA methyltransferase, TrmA family | Protein synthesis | -1.18 | -0.17 | -0.84 | -0.36 |
| PG1097 |  | Mur ligase domain protein/alanine racemase | Cell envelope | -1.71 | -0.21 | -1.43 | -0.20 |
| PG1125 |  | hypothetical protein | Hypothetical protein | -1.29 | -0.51 | -1.25 | -0.29 |
| PG1126 | uraA | uracil permease | Transport and binding proteins | -0.93 | -0.87 | -1.11 | -0.36 |
| PG1136 |  | hypothetical protein | Hypothetical protein | -2.52 | -0.39 | -1.26 | -0.47 |
| PG1137 | porS | porS protein | Unknown function | -3.01 | -0.31 | -1.01 | -1.02 |
| PG1138 | porR | pigmentation and extracellular proteinase regulator | Regulatory functions | -1.46 | -0.33 | -1.05 | -0.31 |
| PG1140 |  | glycosyl transferase, group 2 family protein | Cell envelope | -1.22 | -0.15 | -0.92 | -0.15 |
| PG1141 |  | glycosyl transferase, group 1 family protein | Cell envelope | -1.22 | -0.34 | -0.79 | -0.24 |
| PG1142 |  | exopolysaccharide synthesis-related protein | Unknown function | -1.02 | -0.64 | -1.31 | -0.45 |
| PG1176 |  | ABC transporter, ATP-binding protein, putative | Transport and binding proteins | -1.27 | -0.30 | -1.16 | -0.22 |
| PG1206 |  | hypothetical protein | Hypothetical protein | -1.64 | -0.11 | -1.14 | -0.22 |
| PG1207 |  | hypothetical protein | Hypothetical protein | -1.69 | -0.41 | -1.07 | -0.27 |
| PG1209 |  | hypothetical protein | Hypothetical protein | -0.85 | -0.17 | -1.14 | -0.07 |
| PG1210 |  | peptidase, M24 family protein | Protein fate | -0.88 | -0.40 | -0.82 | -0.17 |
| PG1211 |  | hexapeptide transferase family protein | Unknown function | -1.15 | -0.24 | -1.27 | -0.11 |
| PG1212 |  | TPR domain protein | Unknown function | -0.97 | -0.32 | -1.18 | -0.16 |
| PG1213 | rnhA | ribonuclease H | Transcription | -1.56 | -0.07 | -1.54 | -0.10 |
| PG1282 |  | conserved hypothetical protein | Hypothetical protein | -1.18 | -0.33 | -1.51 | -0.23 |
| PG1294 | feoB-2 | ferrous iron transport protein B | Transport and binding proteins | -1.05 | -0.48 | -0.90 | -0.30 |
| PG1342 | murB | UDP-N-acetylenolpyruvoylglucosamine reductase | Cell envelope | -1.49 | -0.21 | -1.55 | -0.04 |
| PG1343 | lipB | lipoate-protein ligase B | Protein fate | -1.60 | -0.12 | -1.47 | -0.12 |
| PG1351 |  | hypothetical protein | Hypothetical protein | -1.00 | -0.20 | -1.22 | -0.11 |
| PG1359 |  | hypothetical protein | Hypothetical protein | -1.49 | -0.64 | -1.52 | -0.27 |
| PG1404 |  | rhomboid family protein | Unknown function | -1.07 | -0.48 | -1.02 | -0.35 |
| PG1422 | dacB | D-alanyl-D-alanine carboxypeptidase | Cell envelope | -1.28 | -0.51 | -1.93 | -0.31 |
| PG1469 |  | type I restriction-modification system, M subunit, putative | DNA metabolism | -1.32 | -0.74 | -0.87 | -0.80 |
| PG1521 |  | O-succinylbenzoic acid--CoA ligase | Biosynthesis of cofactors, prosthetic groups, and carriers | -1.00 | -0.45 | -1.30 | -0.15 |
| PG1528 |  | conserved hypothetical protein | Hypothetical protein | -1.14 | -0.53 | -1.02 | -0.51 |
| PG1560 | rfbB | dTDP-glucose 4,6-dehydratase | Cell envelope | -1.23 | -0.14 | -0.83 | -0.34 |
| PG1561 | rfbD | dTDP-4-dehydrorhamnose reductase | Cell envelope | -1.39 | -0.04 | -1.30 | -0.15 |
| PG1562 | rfbC | dTDP-4-dehydrorhamnose 3,5-epimerase | Cell envelope | -1.19 | -0.08 | -0.98 | -0.14 |
| PG1577 | nadC | nicotinate-nucleotide pyrophosphorylase | Biosynthesis of cofactors, prosthetic groups, and carriers | -1.02 | -0.20 | -0.93 | -0.09 |
| PG1578 | nadA | quinolinate synthetase complex, subunit A | Biosynthesis of cofactors, prosthetic groups, and carriers | -0.92 | -0.15 | -1.02 | -0.18 |
| PG1588 |  | conserved hypothetical protein | Hypothetical protein | -1.28 | -0.16 | -1.20 | -0.37 |
| PG1594 |  | ComEC/Rec2-related protein | Unknown function | -1.44 | -0.57 | -0.99 | -0.70 |
| PG1598 |  | lipoprotein signal peptidase, putative | Protein fate | -1.07 | -0.53 | -0.97 | -0.42 |
| PG1600 |  | membrane protein, putative | Cell envelope | -2.30 | -0.38 | -2.30 | -0.46 |
| PG1601 |  | biotin--acetyl-CoA-carboxylase ligase | Protein fate | -1.12 | -0.42 | -1.29 | -0.15 |
| PG1608 | mmdB | methylmalonyl-CoA decarboxylase, beta subunit | Energy metabolism | -1.08 | -0.63 | -0.97 | -0.60 |
| PG1609 | mmdC | methylmalonyl-CoA decarboxylase, gamma subunit | Energy metabolism | -1.16 | -0.25 | -1.09 | -0.14 |
| PG1610 |  | hypothetical protein | Hypothetical protein | -1.43 | -0.10 | -1.54 | -0.06 |
| PG1611 |  | hypothetical protein | Hypothetical protein | -1.63 | -0.10 | -1.59 | -0.12 |
| PG1612 | mmdA | methylmalonyl-CoA decarboxylase, alpha subunit | Energy metabolism | -1.15 | -0.27 | -1.16 | -0.26 |
| PG1618 |  | conserved hypothetical protein | Hypothetical protein | -1.01 | -0.54 | -0.82 | -0.47 |
| PG1619 |  | biotin synthesis protein BioC, putative | Biosynthesis of cofactors, prosthetic groups, and carriers | -0.88 | -0.60 | -0.89 | -0.53 |
| PG1648 |  | RelA/SpoT family protein | Cellular processes | -1.05 | -0.50 | -1.30 | -0.26 |
| PG1676 | pckA | phosphoenolpyruvate carboxykinase (ATP) | Energy metabolism | -1.74 | -0.14 | -0.84 | -0.39 |
| PG1696 |  | type II DNA modification methyltransferase, putative | DNA metabolism | -1.21 | -0.42 | -1.17 | -0.18 |
| PG1697 |  | type II restriction endonuclease, putative | DNA metabolism | -1.57 | -0.33 | -1.50 | -0.08 |
| PG1727 | yitL | yitL protein | Unknown function | -1.09 | -0.19 | -0.97 | -0.12 |
| PG1728 |  | cytidine/deoxycytidylate deaminase family protein | Unknown function | -1.21 | -0.27 | -0.81 | -0.21 |
| PG1733 |  | hypothetical protein | Hypothetical protein | -1.34 | -0.30 | -1.48 | -0.22 |
| PG1734 |  | transporter, putative | Transport and binding proteins | -1.17 | -0.55 | -1.27 | -0.47 |
| PG1735 |  | MutT/nudix family protein | DNA metabolism | -0.99 | -0.36 | -0.82 | -0.40 |
| PG1738 |  | hypothetical protein | Hypothetical protein | -1.48 | -0.18 | -0.95 | -0.32 |
| PG1747 |  | ribose 5-phosphate isomerase B, putative | Energy metabolism | -1.30 | -0.12 | -1.30 | -0.12 |
| PG1791 |  | hypothetical protein | Hypothetical protein | -1.07 | -0.42 | -0.89 | -0.31 |
| PG1797 |  | DNA-binding response regulator/sensor histidine kinase | Signal transduction | -1.10 | -0.41 | -0.83 | -0.26 |
| PG1812 |  | 2-oxoglutarate oxidoreductase, alpha subunit | Energy metabolism | -1.22 | -0.16 | -0.98 | -0.20 |
| PG1827 |  | RNA polymerase sigma-70 factor, ECF subfamily | Transcription | -1.35 | -0.42 | -0.84 | -1.23 |
| PG1842 |  | acetyltransferase, GNAT family | Unknown function | -1.17 | -0.49 | -0.85 | -0.77 |
| PG1849 | recN | DNA repair protein RecN | DNA metabolism | -1.23 | -0.21 | -1.27 | -0.21 |
| PG1850 |  | hypothetical protein | Hypothetical protein | -1.18 | -0.28 | -1.33 | -0.23 |
| PG1851 | coaBC | dfp protein | Biosynthesis of cofactors, prosthetic groups, and carriers | -1.20 | -0.12 | -1.09 | -0.10 |
| PG1852 |  | exonuclease | Unknown function | -0.85 | -0.11 | -0.79 | -0.14 |
| PG1853 | dnaN | DNA polymerase III, beta subunit | DNA metabolism | -0.96 | -0.31 | -0.85 | -0.23 |
| PG1857 |  | hypothetical protein | Hypothetical protein | -2.79 | -0.23 | -1.20 | -0.79 |
| PG1861 |  | hypothetical protein | Hypothetical protein | -0.99 | -0.31 | -1.51 | -0.25 |
| PG1862 |  | hypothetical protein | Hypothetical protein | -1.05 | -0.23 | -1.11 | -0.31 |
| PG1863 |  | hypothetical protein | Hypothetical protein | -1.00 | -0.27 | -0.92 | -0.13 |
| PG1864 |  | leucine-rich protein | Unknown function | -1.21 | -0.28 | -1.16 | -0.15 |
| PG1871 |  | hypothetical protein | Hypothetical protein | -1.06 | -0.36 | -0.83 | -0.28 |
| PG1878 | cysS | cysteinyl-tRNA synthetase | Protein synthesis | -1.00 | -0.39 | -0.90 | -0.20 |
| PG1879 |  | conserved hypothetical protein | Hypothetical protein | -1.16 | -0.30 | -1.05 | -0.12 |
| PG1880 |  | glycosyl transferase, group 2 family protein | Cell envelope | -1.45 | -0.37 | -1.73 | -0.27 |
| PG1881 |  | hypothetical protein | Hypothetical protein | -1.17 | -0.35 | -0.97 | -0.10 |
| PG1888 |  | conserved hypothetical protein | Hypothetical protein | -1.02 | -0.44 | -0.81 | -0.19 |
| PG1892 |  | hypothetical protein | Hypothetical protein | -0.86 | -0.49 | -0.86 | -0.36 |
| PG1895 |  | hypothetical protein | Hypothetical protein | -1.63 | -0.37 | -1.14 | -0.65 |
| PG1900 |  | conserved hypothetical protein | Hypothetical protein | -0.92 | -0.14 | -1.16 | -0.33 |
| PG1965 |  | voltage gated chloride channel, authentic frameshift | Transport and binding proteins | -1.02 | -0.83 | -1.26 | -0.45 |
| PG1983 |  | CRISPR-associated protein, TM1791 family | Mobile and extrachromosomal element functions | -0.83 | -0.51 | -0.93 | -0.20 |
| PG1984 |  | CRISPR-associated protein, TM1791.1 family | Mobile and extrachromosomal element functions | -0.85 | -0.47 | -1.17 | -0.15 |
| PG1985 |  | CRISPR-associated protein, TM1792 family | Mobile and extrachromosomal element functions | -1.34 | -0.33 | -1.62 | -0.12 |
| PG1986 |  | CRISPR-associated protein, Cmr3 family (cmr3) | Mobile and extrachromosomal element functions | -1.19 | -0.15 | -1.76 | -0.11 |
| PG1987 |  | CRISPR-associated protein, TM1794 family | Mobile and extrachromosomal element functions | -1.09 | -0.51 | -1.22 | -0.33 |
| PG1988 |  | hypothetical protein | Hypothetical protein | -1.50 | -0.53 | -1.49 | -0.40 |
| PG1989 |  | hypothetical protein | Hypothetical protein | -1.72 | -0.41 | -2.01 | -0.22 |
| PG1993 | uvrC | excinuclease ABC, C subunit | DNA metabolism | -0.92 | -0.85 | -1.19 | -1.21 |
| PG1994 | dtd | D-tyrosyl-tRNA(Tyr) deacylase | Protein synthesis | -1.13 | -0.59 | -1.35 | -0.27 |
| PG1995 |  | conserved hypothetical protein | Hypothetical protein | -1.12 | -0.54 | -1.37 | -0.28 |
| PG2015 | cas4 | CRISPR-associated protein, Cas4 | Mobile and extrachromosomal element functions | -1.34 | -0.49 | -1.22 | -0.32 |
| PG2016 | cas3 | CRISPR-associated protein, Cas3 | Mobile and extrachromosomal element functions | -1.18 | -0.58 | -0.97 | -0.48 |
| PG2017 |  | hypothetical protein | Hypothetical protein | -1.79 | -0.51 | -1.78 | -0.39 |
| PG2018 |  | hypothetical protein | Hypothetical protein | -1.07 | -0.65 | -1.72 | -0.31 |
| PG2019 |  | hypothetical protein | Hypothetical protein | -1.13 | -0.24 | -1.52 | -0.09 |
| PG2020 |  | CRISPR-associated protein, TM1814 family | Mobile and extrachromosomal element functions | -1.01 | -0.26 | -0.95 | -0.20 |
| PG2032 | priA | primosomal protein n' | DNA metabolism | -0.86 | -1.65 | -1.19 | -1.21 |
| PG2047 |  | helicase, putative | Unknown function | -0.85 | -0.50 | -1.03 | -0.46 |
| PG2061 | folA | dihydrofolate reductase | Biosynthesis of cofactors, prosthetic groups, and carriers | -1.14 | -0.08 | -1.29 | -0.23 |
| PG2072 |  | UvrD/REP helicase domain protein | Unknown function | -0.98 | -0.38 | -1.13 | -0.14 |
| PG2087 |  | conserved hypothetical protein | Hypothetical protein | -1.54 | -0.31 | -1.41 | -0.26 |
| PG2088 | msrA | peptide methionine sulfoxide reductase | Protein fate | -1.45 | -0.08 | -1.26 | -0.14 |
| PG2089 |  | hypothetical protein | Hypothetical protein | -1.27 | -0.30 | -1.11 | -0.31 |
| PG2095 |  | lipoprotein, putative | Cell envelope | -0.92 | -0.40 | -0.83 | -0.34 |
| PG2096 |  | conserved domain protein | Hypothetical protein | -0.86 | -0.40 | -0.89 | -0.18 |
| PG2133 |  | lipoprotein, putative | Cell envelope | -1.14 | -0.29 | -1.34 | -0.51 |
| PG2134 |  | lipoprotein, putative | Cell envelope | -0.99 | -0.35 | -1.11 | -0.25 |
| PG2145 |  | polysaccharide deacetylase | Energy metabolism | -1.59 | -0.20 | -1.35 | -0.43 |
| PG2159 |  | protoporphyrinogen oxidase | Biosynthesis of cofactors, prosthetic groups, and carriers | -2.25 | -0.17 | -2.20 | -0.30 |
| PG2163 | surE | stationary-phase survival protein SurE | Cellular processes | -1.66 | -0.09 | -0.92 | -0.27 |
| PG2173 | omp28 | outer membrane lipoprotein Omp28 | Cell envelope | -0.93 | -0.22 | -0.88 | -0.12 |
| PG2223 |  | glycosyl transferase, group 2 family protein | Cell envelope | -1.15 | -0.48 | -0.89 | -0.49 |
